# Fluorescent label-free quantitative detection of nano-sized bioparticles using a pillar array

**DOI:** 10.1038/s41467-018-03596-z

**Published:** 2018-03-28

**Authors:** Kerwin Kwek Zeming, Thoriq Salafi, Swati Shikha, Yong Zhang

**Affiliations:** 1 Department of Biomedical Engineering, Faculty of Engineering, National University of Singapore, Singapore, 117583 Singapore; 2 NUS Graduate School for Integrative Sciences and Engineering, National University of Singapore, Singapore, 117456 Singapore

## Abstract

Disease diagnostics requires detection and quantification of nano-sized bioparticles including DNA, proteins, viruses, and exosomes. Here, a fluorescent label-free method for sensitive detection of bioparticles is explored using a pillar array with micrometer-sized features in a deterministic lateral displacement (DLD) device. The method relies on measuring changes in size and/or electrostatic charges of 1 µm polymer beads due to the capture of target bioparticles on the surface. These changes can be sensitively detected through the lateral displacement of the beads in the DLD array, wherein the lateral shifts in the output translates to a quantitative measurement of bioparticles bound to the bead. The detection of albumin protein and nano-sized polymer vesicles with a concentration as low as 10 ng mL^−1^ (150 pM) and 3.75 μg mL^−1^, respectively, is demonstrated. This label-free method holds potential for point-of-care diagnostics, as it is low-cost, fast, sensitive, and only requires a standard laboratory microscope for detection.

## Abstract (web summary)

The detection and quantification of bioparticles is important to a range of applications including disease diagnostics. Here, the authors present a fluorescent label-free detection method using deterministic lateral displacement and demonstrate its use in detecting a range of proteins and vesicles.

## Introduction

Disease diagnosis requires identification and quantification of various bioparticles such as DNA, RNA, proteins, virus, exosomes, and bacteria. Current clinical laboratories use well-established sandwich assay, PCR, gel electrophoresis, and flow-cytometry methods for detection of these bioparticles^1,2^. However, these methods use fluorescent labels that increase detection cost and complexity by reliance on expensive optical systems and involvement of multiple sample processing steps requiring minimum sample volumes. Thus, fluorescent label-free bioparticle detection gains traction as an alternative means in disease diagnosis.

Technological advancement in label-free methods using microcantilever^3^, surface-enhanced Raman scattering (SERS)^4^, surface plasmon resonance (SPR)^5–7^, magnetic beads^8^, electrochemical detection^9^, and quartz crystal microbalance^10^ provide real-time information on bioparticle interactions, resulting in greater understanding of biochemical functions, drug interactions, and sensitive quantification of these bioparticles. The biosensor tracks changes in biophysical interactions of binding events, mass changes, refractive index or chemical reactions, and transduces the information as mechanical, electrical, or optical signals, and have shown detection of proteins down to femtomolar levels. However, these techniques often require precision engineering of nano-features, complex optical setups, secondary antibodies in sandwich assays, novel nanoprobes (e.g., graphene oxide, carbon nanotubes, and gold nanorods) or additional amplification step such as aggregation of nanoparticles to reduce the limit of detection (LOD)^11^.

Deterministic lateral displacement (DLD) pillar array platforms have been used for size-sensitive separation of circulating tumour cells to bioparticles such as DNA and exosomes^12–15^. For a fixed critical DLD cut-off size (*D*_c_), larger particles get displaced laterally relative to particles smaller than the *D*_c_^16,17^. To separate nano-sized particles, it is challenging and costly to operate due to nanofabrication, precision injection of sample, and low throughput due to the small gap size^18^. So far, DLD research has mainly been focused on bioparticle separation and potential use of this technique for detection has not been extensively explored^19,20^.

Here, a fluorescent label-free method for sensitive detection of nano-sized proteins and polymer vesicles using a DLD pillar array with micrometer-sized features is demonstrated. Bioparticles of interest are captured or adsorbed onto polymer microbeads with specific ligands and detected quantitatively based on lateral displacement of the microbeads in the pillar array. Two domains exist for this bioparticle detection phenomenon: for small bioparticles, electrostatic interactions dominate, and for large bioparticles, physical particle size increase has a dominant role. The detection is performed through lateral displacement changes as a result from the modulation of microbead surface charge or size induced by the adsorption of bioparticles. The extent of the lateral displacement can be correlated to the amount of bioparticles in the sample. Using this bioparticle-on-bead method, changes in lateral shift correlating to the bioparticle concentrations can be sensitively discriminated. The detection of albumin proteins and nano-sized polymer vesicles with a concentration as low as 10 ng mL^−1^ (150 pM) and 3.75 μg mL^−1^, respectively is demonstrated. This work sets the precedent for sensitive detection and quantification of biological particles via sensitive changes in size or electrostatic interactions of the microbead carrier on DLD. We pushed the boundaries of DLD and applied our model for fluorescent label-free detection of nano-sized proteins and polymer vesicles, which open opportunities for low-cost medical diagnosis, liquid biopsy, and detection of biologically relevant DNA, RNA, exosomes, viruses, and proteins.

## Results

### Electrostatic influence on particle–DLD interactions

**Fig. 1:**
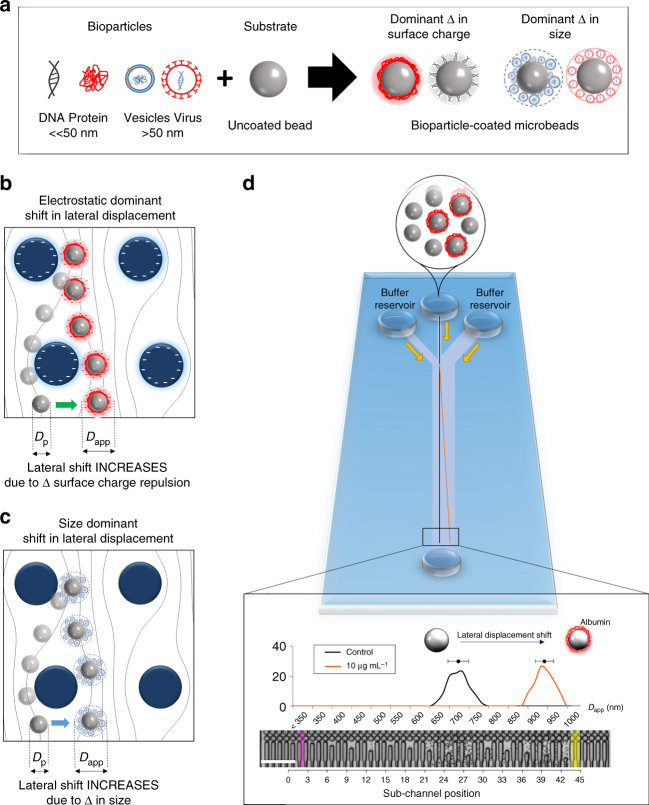
DLD-based detection of bioparticles using coated micro-bead substrate. **a** Adhering bioparticles on to the surface of micro-beads causing overall change in surface charge and size of bioparticle-microbead conjugates. Two mechanisms proposed to increase the apparent diameter (*D*_app_) for detection using **b** electrostatic charge repulsion depicted by the negatively charged pillars and bead, and **c** size increase of the bioparticle-microbead conjugate. **d** Shows the DLD device schematics, which can sensitively detect a lateral shift of a 10 µg mL^−1^ albumin coated with a mixture of uncoated 1 µm PS-COOH beads. Lateral shifts in the DLD output spectrum can be correlated to the corresponding *D*_app_. The black line shows the input position of the microbead sample, magenta band represents where *D*_app_ = 350 nm (0.35 µm), and yellow band represents the maximum displaced spectrum of 1000 nm (1 µm). Scale bar is 50 µm

**Fig. 2:**
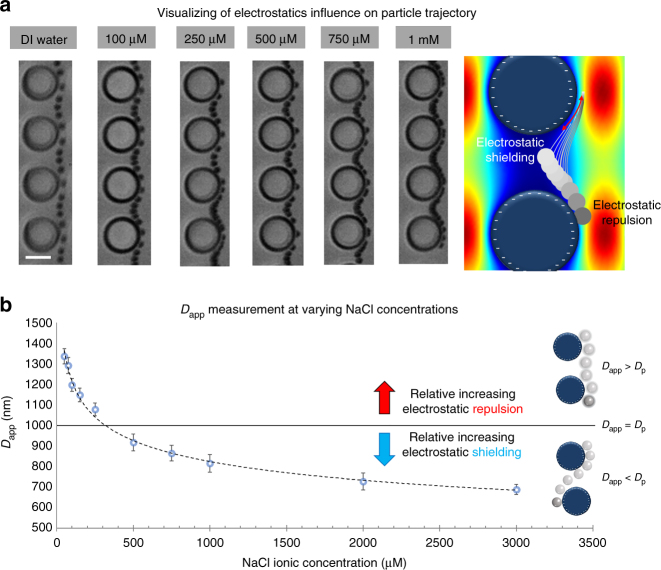
Visualization of electrostatic dominant lateral shift phenomena. **a** Shows a tracked motion of 1 µm PS beads within a DLD segment with predicted *D*_app_ to be > 700 nm and COMSOL computation of various trajectory taken by a 1 µm particle due to shifts in stream lines. Scale bar is 5 µm. **b** NaCl buffer concentration modulates the electrostatic interactions and thus changes the *D*_app_. A decrease in ionic concentration results in a larger electrostatic repulsion and thus a positive increase in lateral shift where *D*_app_ > *D*_p_. Conversely, the particle will appear smaller than *D*_p_ due to increased electrostatic shielding for higher ionic concentrations. The error bars show the SD from at least 50 sample counts of beads

Electrostatic forces in DLD are non-trivial and can significantly influence particle-DLD interactions^21^ (Fig. 1b). We investigated these effects using a × 600 magnification and high-speed capture of 1000 fps, and different particle positions were experimentally tracked and superimposed (Fig. 2a). The DLD segment used for this capture had a gap of 4 µm and gradient of 0.75° resulting in a *D*_c_ of 700 nm and the interaction between 1 µm particle and the DLD pillar is ensured. The difference between different ionic media is evident in the particle motion and distance between particle positions. The electrostatics force repels the beads from the pillar at low ionic concentration, which displace the beads into a streamline further away from the pillar. The simulations show that small shifts in particle streamlines could drastically change the curvature of motion of particle. We also measured the mean flow velocity of these particles and found that increasing the ionic concentration reduces the average particle flow velocity (Supplementary Fig. 1). This confirms that at lower ionic concentrations, electrostatic interactions shifts the beads into a streamline away from the pillar resulting in an increase in velocity and shift in lateral displacement.

To evaluate the extend of electrostatic lateral shift in *D*_app_, a baseline reference curve for DLD device was developed by characterising *D*_app_ using DLD-S1 device under control conditions of native poly-dimethylsiloxane (PDMS) surface with 1 µm polystyrene (PS) National Institute of Standards and Technology (NIST) beads and NaCl solutions of different concentrations (Fig. 2b). The curve represents the mean *D*_app_ size based on various conditions of separation spectrums (Supplementary Fig. 2).

At ~ 350 µM NaCl, *D*_app_ = *D*_p_ where the stipulated particle size is comparable to the *D*_app_. NaCl concentrations lower than 350 µM yield a greater *D*_app_ due to increase in electrostatic repulsion, whereas higher NaCl concentrations would mean an increase in surface charge shielding and smaller *D*_app_. It is important to note that the size of the particle do not change, rather the changing electrostatic interactions shifts the particle into different streamlines in DLD resulting in sensitive changes in apparent size. Although the actual size of particle does not change, the cause of the smaller *D*_app_ influence has not yet been investigated, a likely reason is due to the attraction of particle towards the pillar via hydrophobic interaction when the electrostatic effect is shielded^22^. This is supported by the increase of bead adhesion on pillar at high ionic concentration above 100 mM^23^. The *D*_app_ curve serves as a baseline reference for comparison with various parameters of surface charge, particle surface, and different buffers to be tested and optimized for bioparticle detection.

### Optimizing surface charge parameters for DLD separation

**Fig. 3:**
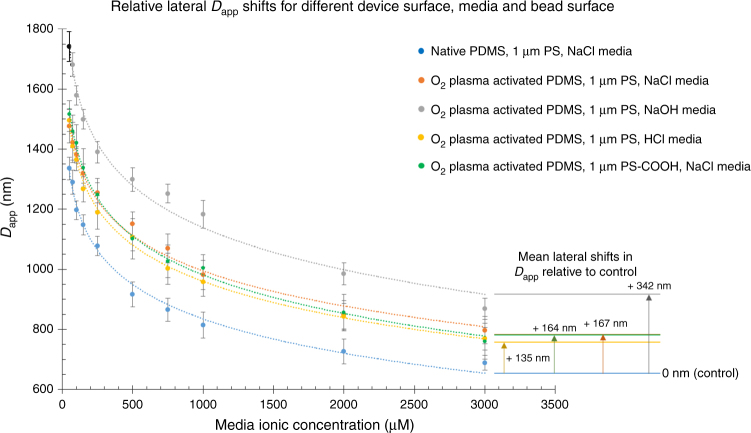
Parameters governing surface charge influence on the lateral *D*_app_ shift. Plot of the relative lateral *D*_app_ shift for different device surface, bead surface, and buffer media at different ionic concentrations. The shift is calculated relative to native PDMS control. A positive shift would mean an increase in *D*_app_ across all ionic concentration levels. Each data point was plotted based on at least 50 sample counts and the error bar represent the SD of the sample *D*_app_

Three parameters are used to experimentally investigate the surface charge effects on *D*_app_ shifts namely, PDMS device surface charges, bead surface charges, and pH of the fluid media. Figure 3 summarizes the effects of various parameters on *D*_app_ curve plots at different ionic concentrations. Despite large difference in *D*_app_ shifts across different parameters, there is minimal difference in bead sizes as observed with transmission electron microscopy (TEM) and dynamic light scattering (DLS) (Supplementary Fig. 3).

Figure 3 shows the measured mean lateral shifts in *D*_app_ between various DLD experimental parameters and the reference standard curves of native PDMS, 1 µm PS NIST beads, and NaCl solution. Using the control *D*_app_ curve as the reference, the shift in *D*_app_ curves in the DLD-S1 can be measured to characterize the influence of these parameters (Supplementary Fig. 4). The positive ( + ) or negative ( – ) shift of *D*_app_ is made relative to the control *D*_app_. The plasma treated surface increases the mean *D*_app_ by + 164 nm compared with native PDMS. Plasma activation increases the number of SiO^−^ surface groups and creates a highly negatively charged PDMS surface with isoelectric point of pH 2 to pH 5^24–26^. By exposing plasma activated surface to different pH solutions, the *D*_app_ values change dramatically as the different pH influences the magnitude and ionization of the surface groups^27^. The use of NaOH ranging from pH 9.7 to 11.5 (50–3000 µM) would alter the electrostatic interactions by increasing the mean *D*_app_ to + 342 nm compared with pure native PDMS in NaCl solution. The use of HCl on the contrary reduces the electrostatic influence of the highly-charged plasma activated PDMS surface^25^. This is expected as the association of H^+^ to the surface of the DLD device would result in reduction of negatively charged groups, thus reducing electrostatic interactions to + 135 nm mean *D*_app_. It is important to note that the surface charge of plasma-treated PDMS is still negative as it is still beyond its isoelectric point.

Different bead surfaces namely PS-, PS-carboxylated (COOH)-, and poly-allylamine hydrochloride (PAH)-coated beads were also tested. Using the same plasma-activated surface, COOH beads displayed a mean *D*_app_ shift of + 167 nm, which is similar to plain PS beads at + 164 nm. Further measurements of colloidal zeta-potential suggest similar colloidal stability and potential of – 21.4 mV for PS and – 29.5 mV for PS-COOH in deionised (DI) water. To induce a positively charged bead, the PS-COOH bead was coated with positively charged PAH which would physically adsorb to the negatively charged bead. The zeta potential measurement shows the PAH coating results in the + 43 mV surface zeta potential in DI water. The beads were flowed into the DLD setup and as expected, the positively charged beads attracted to the negatively charged DLD device surface at the entrance of the reservoir and could not enter the DLD pillar region (Supplementary Fig. 5). Therefore, electrostatic interactions significantly influence particle separation in DLD device and ensuring charge repulsion of particle-DLD surface is necessary.

### Florescent label-free detection of protein coated beads

A protein-coated bead is predicted to change the surface properties of the bead substrate which will result in a change in lateral *D*_app_ shift. The amount of *D*_app_ shift is hypothesized to correlate to the amount of proteins on the bead surface. Native albumin protein has a hydrodynamic radius of 3.7 nm and a protein coated bead would increase the theoretical hydrodynamic size of the bead by at most 10 nm^28^. This size difference is lower than the resolution of our device (Supplementary Note 1 and Supplementary Fig. 6). However, using the particle-pillar electrostatic effects, these differences could be easily detected in the DLD setup by quantifying the changes in the *D*_app_ of beads. The DLD-S2 device has a higher theoretical resolution limit of ~ 10 nm and would be used in this study to increase the sensitivity of electrostatic-induced interaction.

**Fig. 4:**
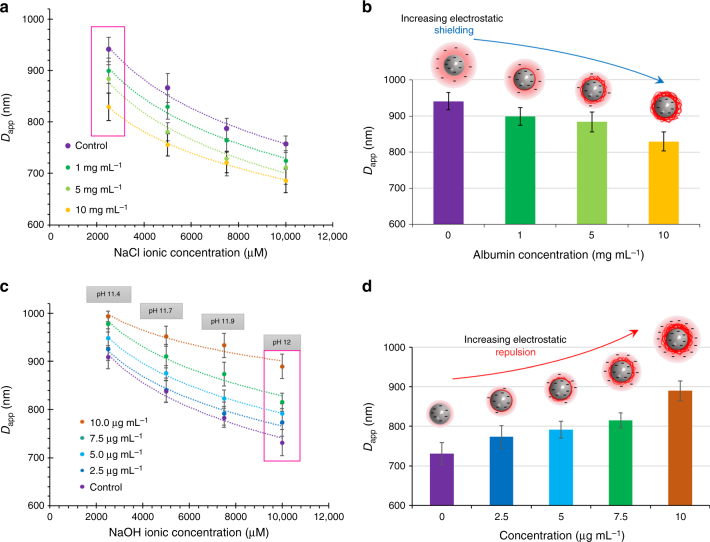
Optimizing albumin concentration detection range and electrostatic interactions. **a** Shows detection of proteins in the mg mL^−1^ range using varying NaCl concentrations as the media. The greatest difference in mean lateral *D*_app_ shifts is highlighted in the magenta box and shown in **b** where increasing protein concentration shields the surface charges on the beads. **c** Using NaOH alkaline solution, detection of proteins within the µg ml ^−1^ range was performed and the greatest difference of the mean lateral shifts is shown in pH 12 NaOH media (within the magenta box). **d** Shows the *D*_app_ of different coated beads in a NaOH pH 12 media. All data point comprises of the distribution of at least 50 beads with the error bar representing the SD of the calculated *D*_app_

PS microbeads were suspended in different concentrations of albumin solutions to form an albumin coat on the bead with DI water as the adsorption media. The beads suspended in proteins of concentration range from 1 to 10.0 mg mL^−1^ showed decreasing *D*_app_ (Fig. 4a). The zeta-potential of the beads in NaCl solutions was measured and it was found that the surface charges were shielded (Supplementary Fig. 7). This result corresponds to a decrease in mean *D*_app_ for an albumin coated bead since electrostatic charges are muted due to the albumin coat in NaCl solution. The use of NaCl in the range of 2000 mM elicited the largest difference between the different protein concentrations (Fig. 4b and Supplementary Fig. 8a).

We tested the effects of using alkaline pH solutions on the increase in electrostatic interactions and detection sensitivity. At high pH, the albumin protein would unfold and the charge density would have increased significantly due to disassociation of H^+^ charged groups from the albumin^29^. The impact of pH of medium on protein-coated beads' apparent diameter as compared to the *D*_app_ in NaCl solution showed a significant increase of approximately 400-fold in sensitivity of protein coat detection (from 1 mg mL^−1^ to 2.5 µg mL^−1^). Figure 4c shows the separation of 2.5–10 µg mL^−1^ albumin protein-coated COOH beads in alkaline NaOH solution of concentration 2.5–10.0 mM (pH 11.4–12). At 2.5 µg mL^−1^ of albumin, the protein coat formed on COOH beads have a mean *D*_app_ difference of 18 nm. However, at 5.0 µg mL^−1^, the mean difference has increased to 44 nm, which shows difference in peaks across all alkaline pH. The most significant observable difference is at 10 µg mL^−1^ protein concentration where there is a relatively large increase of 127 nm in *D*_app_ of coated beads compared to the non-coated beads in alkaline pH 12 NaOH solution (Fig. 4d and Supplementary Fig. 8b). On the contrary, low pH solutions were tested and it was found that even at 1 mM HCl, the albumin-coated beads started to stick non-specifically to the device (Supplementary Fig. 9). This is because the charges on the beads have changed from negative to positive for pH 4. This also indirectly confirms the presence of albumin on the beads as the pI of albumin is ~ 4.7^30^. This charge inversion facilitates the electrostatic attraction force between the positively charged beads and surface of negatively charged microchannels.

**Fig. 5:**
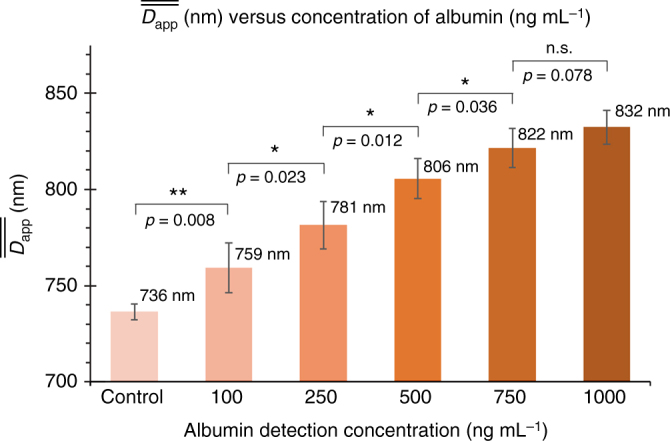
The detection of albumin in the ng mL^−1^ range. The bar graph represents the different $\overline{\overline {{{D}}_{{\mathrm{app}}}}}$, mean of mean *D*_app_, for various protein concentration. The control beads were not exposed to albumin, while the albumin concentrations for detection were ranged from 100 to 1000 ng mL^−1^. Four sets of experiments were independently performed and the mean of mean *D*_app_ values were analysed (*n* = 4) with SD as the error bars. Independent two-sample *t*-test is used for the statistical analysis where *p < 0.05, **p < 0.01, and n.s. = not significant

An optimized adsorption albumin protocol was performed using pH 5.5 2-morpholinoethanesulfonic acid (MES) buffer and lower concentrations of the beads. This, combined with the use of pH 12 NaOH solution for DLD separation, results in a significant decrease in the limit of albumin detection concentration (Fig. 5 and Supplementary Fig. 10). Four independent sets of samples were tested using four PDMS devices and the mean *D*_app_ of the four samples were averaged and plotted as $\overline{\overline {{D}_{{\mathrm{app}}}}}$ in Fig. 5 for various concentration of protein adsorption ranging from 100 to 1000 ng mL^−1^ (Supplementary Fig. 11). The results showed that we could detect as low as 100 ng mL^−1^ of albumin, which corresponds to approximately 1.5 nM of albumin using this label-free approach. The $\overline{\overline {{D}_{{\mathrm{app}}}}}$ of 750 ng mL^−1^ and 1000 ng mL^−1^ were 822 and 832 nm, respectively, which did not yield a significant difference of a  10 nm *D*_app_ change, which is at the limits of resolution for the DLD-S2 device.

**Fig. 6:**
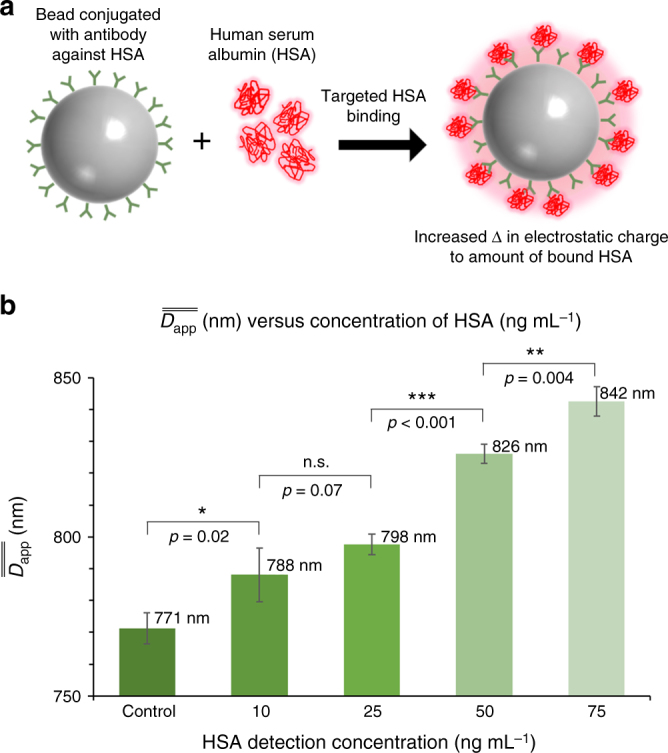
Detection of HSA using antibody coated beads. **a** Schematics showing the detection of human serum albumin with the capture of antibody for higher detection sensitivity. **b** Three sets of readings were performed for the detection of HSA from 10 to 75 ng mL^−1^ (*n* = 3). $\overline{\overline {{D}_{{\mathrm{app}}}}}$ represents the mean of mean *D*_app_ for the three data sets with SD as the error bars (Supplementary Fig. 13). Independent two-sample *t*-test is used for the statistical analysis. Where *p < 0.05, **p < 0.01, ***p < 0.001, and n.s. = not significant

To ensure specific binding to proteins and higher detection sensitivity, beads conjugated with human serum albumin (HSA) antibody were used to detect the presence of HSA (Fig. 6a). The binding of HSA to the antibody was confirmed using fluorescence labelled secondary antibody binding (Supplementary Fig. 12). The limits of detection of HSA using antibody testing were found to be 10 times more sensitive, ranging from 10 to 75 ng mL^−1^, than the physical adsorption of albumin on 1 µm bead. Similarly, *n* = 3 independent sets of readings were performed using three PDMS device and the corresponding $\overline{\overline {{D}_{{\mathrm{app}}}}}$ detection range for 10–75 ng mL^−1^ of HSA protein were 771–842 nm, respectively (Fig. 6b and Supplementary Fig. 13). The $\overline{\overline {{D}_{{\mathrm{app}}}}}$ of 25 ng mL^−1^ of HSA is not statistically significant compared with 10 ng mL^−1^ as the $\overline{\overline {{D}_{{\mathrm{app}}}}}$ difference is ~ 10 nm, which is close to the LOD of the DLD-S2 device. The detection was performed under NaOH pH 12 for comparison with earlier studies on albumin adsorption to bead. The use of antibody-conjugated beads increases the specificity of protein binding and sensitivity of detection to as low as 150 pM. This method of protein detection does not use fluorescence label, secondary antibody or nanoparticle aggregation methods and is comparable to existing label-free protein detection such as SERS, SPR, or microcantilevers^31,32^.

### Fluorescence label-free detection of vesicle on DLD

Extracellular vesicles (EVs) have importance in intercellular communication, regeneration, and transport.^33–37^ Furthermore, integral proteins present in the membrane of EVs have significant roles in mediating these communications and have emerged as biomarkers for exosomes in various pathological and normal conditions.^38–43^ Thus, the detection of EVs and its membrane proteins is crucial for disease diagnosis. Owing to advantages of mechanical stability and membrane tunability, polymer vesicles were chosen over lipid vesicles for incorporating the membrane proteins for the current study.^44–46^ BD21 vesicles with a TEM size range of 132 ± 31 nm were prepared and characterised for size, shape, and surface charge (Methods and Supplementary Fig. 14).

Similar to protein detection, these nanovesicles were adsorbed onto the surface of the beads and the detection range was found to be between 0.32 and 2.5 mg mL^−1^ under 0.1 × phosphate buffered saline (PBS) media (Supplementary Figs. 14 and 15, and Supplementary Note 2). To confirm that the lateral displacement is dominantly driven by the size increase instead of charge, beads were coated with dissolved vesicles. The dissolved vesicles were prepared via detergent dissolution which resulted in the size of ~ 8 nm and similar surface charge compared with the undissolved vesicle. It was observed that the apparent diameter of the beads coated with dissolved vesicle is similar to the uncoated beads (Supplementary Fig. 16).

To further enhance the detection specificity and sensitivity of nano-vesicles, primary antibodies conjugated beads were used to bind to polymer nano-vesicles reconstituted with Aquaporin-1 proteins (Aqp1). As most EVs contain surface markers and proteins, Aqp1 was reconstituted onto polymer vesicles to demonstrate the detection by antibody coated beads in DLD device based on change in bead size. Aqp1 is a membrane pore protein which allows the permeability of water and was used as a model protein for detection of nano-vesicles.^47,48^ The incorporation of Aqp1 membrane protein to vesicles was done by widely used detergent-mediated reconstitution method that involves vesicle dissolution using detergent and protein reconstitution with removing the detergent using biobeads (Methods). It is important to note that the reconstituted vesicles are smaller compared to the original size of vesicles which could be due to a faster rate of detergent removal (Supplementary Fig. 17)^49^. The concentration of reconstituted Aqp1 vesicles were then assessed by Bicinchoninic Acid (BCA) assay (Supplementary Fig. 18), whereas its functionality was shown by the shrinking of Aqp1 vesicles upon gradual exposure to hyperosmotic sucrose solution compared with vesicles without Aqp1 (Supplementary Table 1).

**Fig. 7:**
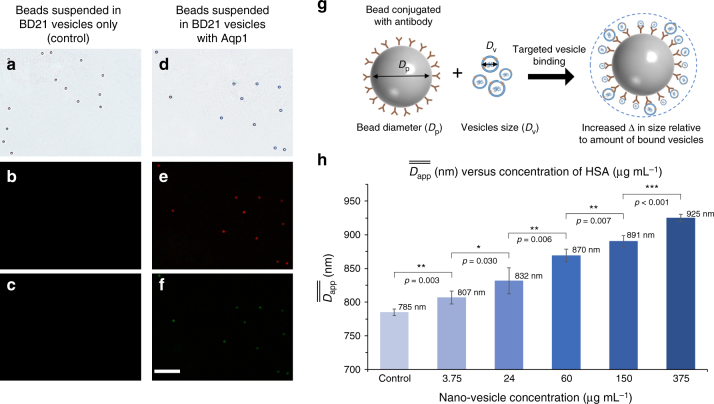
Vesicle detection using antibody immobilised beads. PS-COOH beads (1 µm) conjugated with Aquaporin-1 (Aqp1) antibody incubated with BD21 vesicles without Aqp1 protein showing no binding of vesicles **a**–**c**. The specific binding of BD21 vesicles containing Aqp1 protein on the anti-Aqp1-beads as confirmed by fluorescence from RBOE dye in vesicles in **e**. Fluorescence from Alexa-fluor 488-labelled secondary antibody further confirmed the presence of Aqp1 in vesicles bound to the anti-Aqp1-beads **f**. Scale bar is 10 µm. **g** Schematics showing the detection based on the change in size due to the binding of vesicles onto the antibody-conjugated beads. The buffer media used here is 0.1 × PBS solution. **h** The detection of vesicle concentrations of 3.75–375 µg mL^−1^ was performed with four sets of samples (*n* = 4) with SD as the error bars. Independent two-sample *t*-test is used for the statistical analysis where *p < 0.05, **p < 0.01, and ***p < 0.001

The binding specificity of the BD21 nano-vesicles to the antibody-conjugated beads was confirmed using fluorescent probes (Fig. 7a–f). Interestingly, the DLD vesicle detection through the antibody-based capture of BD21 vesicles showed a 90-fold increase in limits of detection in comparison with the DLD detection with physical adsorption on beads from 0.33 mg mL^−1^ down to 3.75 µg mL^−1^ (Fig. 7 and Supplementary Fig. 14). Four sets of experiments were performed using four DLD-S2 devices to acquire the data and the corresponding analysis (Supplementary Fig. 19). The detection range of these nano-vesicles now span two orders of magnitude from 3.75 to 375 µg mL^−1^ with a $\overline{\overline {{D}_{{\mathrm{app}}}}}$ ranging from 807 to 925 nm under 0.1 × PBS buffer (Fig. 7). At this ionic concentration, the electrostatic interactions are muted and changes in the bead mean *D*_app_ size is correlated to the increase in the amount of vesicles bound to the antibody-conjugated bead. At low vesicle concentrations, the sparsely bound vesicles hardly change the average diameter of the bead, whereas at 375 µg mL^−1^, the size of the bead increases by 140 nm compared with the control. This concentration is within the detection range of some exosomes isolated from physiological conditions, which can vary from several µg mL^−1^ ^50,51^ down to ng mL^−1^ ^52^ depending on the source of the exosome (plasma or serum), its cells type, pathological status (healthy or diseased), and purpose.

## Discussion

Using albumin and polymer vesicle as proof of concept, we demonstrated a fluorescent label-free method for detection of nano-sized albumin proteins and vesicles using a PDMS DLD pillar array with micrometer-sized features. The advantages of this method are multi-fold. Fabrication of such a pillar array with micrometer-sized features is much less challenging as compared with the devices that require nanofabrication. The fluid flow does not require high pressures and the detection can be easily performed using standard bright-field bench-top microscopes^18^. The attachment of bioparticles on microbeads also significantly reduces the effect of diffusion. Specific ligands can be immobilized on different microbeads to capture different bioparticles of interest. Moreover, the electrostatic interactions between surface proteins and DLD pillars could be modulated in real-time using different buffer ionic concentrations and the resultant lateral shift of the microbeads can be used to detect different amounts of proteins^21^. The detection of HSA proteins on beads via electrostatics dominant change in DLD has a LOD of 10 ng mL^−1^, whereas detection of polymer vesicles based on particle size change has a detection limit of 3.75 µg mL^−1^. The vesicle membrane protein detection by antibody-coated beads in DLD device can be further extended to specific detection of EVs such as exosomes based on their respective membrane proteins.

We have demonstrated that the fluorescence label-free method for nano-sized bioparticle detection is inexpensive, sensitive and only requires a standard laboratory microscope for the measurement of bead lateral position in the DLD PDMS device. Furthermore, with a 50 fps capture framerate, it is possible to integrate the detection onto portable imaging solutions and hold great potential for use in point of care diagnostics.

## Methods

### Device design

Using Eq. 1, two DLD devices were designed with incremental step resolution (Supplementary Fig. 6). DLD System 1 (DLD-S1) was designed for high-dynamic range of *D*_app_ and DLD System 2 (DLD-S2) was designed for high resolution displacement. DLD-S1 has a 100 nm *D*_app_ increment measurement and DLD-S2 is 50 nm. The two systems also have different sensitivity for separation of 1 µm bead substrates in ionic buffer. DLD system 1 (DLD-S1) has dynamic range from 1 µM to 3 mM, whereas DLD system 2 (DLD-S2) has greater range of 0.5–150 mM. These DLD devices have 14 DLD segments connected in series with gap sizes fixed at 4 µm for DLD-S1 and 2 µm for DLD-S2. This results in a particle size resolvable quasi-resolution of 34 nm for DLD-S1 and 17 nm for DLD-S2. The input sample stream is sandwiched by two buffer streams to a width of a single input channel.

### Electrostatic and size dominant separation in DLD

DLD is a robust label-free microfluidics particle separation technique pioneered by Huang et al^13^. This technique uses pillar array with certain gap which is tilted in an angle and generates unique number of streamline between the gap that can laterally displace particle above the critical diameter. This *D*_c_ of the separation is influenced by the gap between pillars and the row shift fraction. Davis et al.^16,53^ proposed an empirical formula for DLD array.^54^

1 $${D}_{\mathrm{c}} = 1.4\,{G}\,{\varepsilon }^{0.48}$$

Where ${G}$ is the gap or pore size between pillars and ${\varepsilon }$ is the row shift fraction $({\varepsilon } = {\mathrm{{tan}\theta }})$ when ${\theta }$ is the angle of the gradient. Particles larger than this *D*_c_ will displaced laterally, whereas particles smaller than *D*_c_ flow through the array without any lateral displacement. Therefore, for lateral displacement to occur, the particle diameter (*D*_p_) must be greater than *D*_c_.

Although Eq. 1 determines the particle physical size for separation, it does not account for the influence of electrostatic forces on the particle cut-off size in DLD. We previously determined that electrostatic force effects on DLD separation is non-trivial even for particles as large as 1 µm^21^. Using our DLD device, we can measure the effect of electrostatic forces by changes in the apparent size of the particles (Supplementary Fig. 20).

2 $$D_{{\mathrm{app}}} = D_{{\mathrm{F}} - {\mathrm{EDL}}} + D_{\mathrm{p}}$$

The ${D}_{{\mathrm{F}} - {\mathrm{EDL}}}$ term describes the additional displacement of the particle due to the summation of hydrodynamic and electrostatic forces acting between the DLD pillar and particle. When ${D}_{{\mathrm{F}} - {\mathrm{EDL}}}$ is positive (${D}_{{\mathrm{app}}}$>${D}_{\mathrm{p}}$), the particle appears larger than it physically is in the DLD device and has a greater lateral displacement (Fig. 1). Interestingly, the converse is possible when ${D}_{{\mathrm{F}} - {\mathrm{EDL}}}$ is negative (${D}_{{\mathrm{app}}}$<${D}_{\mathrm{p}}$), and the particle reduces its apparent size resulting in reduced lateral displacement. This does not mean that the electrostatic force becomes attractive, rather the surface charges on pillar and particle surface are being shielded such that the baseline repulsive force in a stable colloidal system (when ${D}_{{\mathrm{app}}}$=${D}_{\mathrm{p}}$) is reduced. ${D}_{{\mathrm{F}} - {\mathrm{EDL}}}$can be approximated from the following equation,

3 $${\mathbf{F}}_{{\mathrm{F}} - {\mathrm{EDL}}} = \frac{{2{\mathrm{\pi }}\lambda _{\mathrm{D}}R}}{{{\varepsilon_0} \varepsilon }}\left( {\left( {\sigma _{\mathrm{p}}^2 + \sigma _{\mathrm{s}}^2} \right)\mathrm{{e}}^{ - 2D_{{\mathrm{F}} - {\mathrm{EDL}}}/\lambda _{\mathrm{D}}} + 2\sigma _{\mathrm{p}}\sigma _{\mathrm{s}}\mathrm{{e}}^{ - D_{{\mathrm{F}} - {\mathrm{EDL}}}/\lambda _{\mathrm{D}}}} \right)$$

in which ${\mathrm{\sigma }}_{\mathrm{p}}$ is particle surface charge, ${\mathrm{\sigma }}_{\mathrm{s}}$ is device surface charge, and ${\mathrm{\lambda }}_{\mathrm{D}}$ is the debye length of the solution. This Debye length (${\mathrm{\lambda }}_{\mathrm{D}}$) depends on the charge (*z*) and ionic concentration (*c*), temperature *(T*), Boltzmann constant ${k}_{\mathrm{b}}$, electron charge (*e*) and Avogadro number (${N}_{\mathrm{A}}$)

4 $$\lambda _{\mathrm{D}} = \left[ {\frac{{N_{\mathrm{A}}{e}^2}}{{{\varepsilon \varepsilon _0} {k_{\rm b}}T}}\mathop {\sum }\limits_{i} z_i^2c_{i}^\infty } \right]^{ - 1/2}$$

It is predicted from the equations that as ionic concentrations of solution decreases, the electrostatic double layer would increase, suggesting greater electrostatic effects from repulsive surface^22^. This increase in repulsive force virtually increase the diameter (${D}_{\mathrm{p}}$) of the particle by the electrostatics double layer force ($D_{{\mathrm{F}} - {\mathrm{EDL}}}$) results in greater lateral displacement of the particle. Fluid flow velocities do affect the separation but it is not very significant and would require increase in excess of 100-fold before there can be an observable effect. Thus, the electrostatic force interaction on particles in DLD can be primarily influence by these three factors—surface charges on the device, particle, and ionic concentration of media^55^.

Therefore, this electrostatic-based displacement can be used for detection of nano-sized biomolecules such as protein or DNA on microbeads, as the presence of biomolecules coat changes the overall surface charge of the microbeads, and hence the electrostatics force and lateral displacement in sensitive DLD.

In contrast, the size-based change is dominant for larger bioparticles coat such as vesicles. The adsorption of the vesicles with a diameter (*D*_v_) of 50–200 nm to the microbead surface increases the overall size of the bead from a diameter (*D*_p_) of 1 µm to *D*_app_ of ~ 1.05–1.4 µm. It is hypothesized that the *D*_app_ is increased as the amount of adsorbed vesicles increases. At lower vesicle concentration, the increase in *D*_app_ is small due to random adsorption of small amount of vesicles on beads, whereas at higher concentration when the bead surface is fully adsorbed with the vesicles, the *D*_app_ reaches saturation, and hence plateaus off. The usage of antibody specific to the membrane protein increases the sensitivity of the vesicle binding, and hence results in lower LOD of the vesicles.

### Device fabrication

Briefly, SU-8 2005 (MicroChem, USA) was used to develop the DLD device negative mould on a 4-inch silicon wafer at a height of 3 µm. The SU-8 was patterned using a hard chrome glass mask (Infinite Graphics, Singapore) on a SUSS-MA8 lithography mask aligner. The final device was fabricated using PDMS cured on the silicon SU-8 mould. The input and output holes were punched and the PDMS device was bonded onto a glass slide using a oxygen plasma treatment for 2 min in the March PX-250 plasma machine. All devices used in this work were fabricated from the same mould to ensure consistency in results.

### Sample and buffer solutions

Three types of bead samples were used: 1 µm NIST PS beads (Bangslab NT15N, USA), 1 µm amine beads (bangslab PA03N, USA), and 1 µm carboxylated beads (Polyscience 17458, USA). These beads were subsequently diluted to the required concentration of 0.1% (w/v) for all separation experiment. Sodium chloride (Sigma S5150, Singapore), sodium hydroxide (Sigma S2770), and hydrogen chloride (Sigma H9892) were prepared as a stock solution of 1 M. Non-ionic Pluronic F-127 (Sigma P2443) was prepared at 1% (w/v), whereas albumin solution (Sigma A9576) was purchased as 30% (v/v) stock solution. The 50 mM MES buffer pH 5 solution was prepared for the optimized protein adsorption on beads. The 1 × PBS solution (Thermofisher 10010023) was used as the stock PBS solution for vesicle detection. All solutions were diluted subsequently in 18.2 MOhm cm Millipore ultra-pure DI water to the required concentration.

### Protein physical adsorption on beads

Total of 3 µL of 2.64% (w/v) carboxylated beads were put into 1 mL of albumin solution with concentration of 1, 0.5, and 0.1 mg ml^–1^, and 10, 7.5, 5, and 2.5 µg mL^−1^, and incubated for 15 min at room temperature with DI water as a buffer for the physical adsorption of albumin to the beads. Subsequently, the beads were washed with centrifugation for three times at 6000 r.p.m. for 3 min each, to get rid of the remaining unabsorbed albumin in the solution. The beads then were diluted to 15 µL for the experiment. The PAH (Sigma 283223) coating were performed by putting 3 µL beads to 0.01% (w/v) PAH in 0.5 M NaCl buffer for 5 min and the solution were washed 3 × to remove unabsorbed PAH solution. The optimized protein adsorption for more sensitive detection was performed using MES buffer and smaller volume of beads. Total of 0.5 µL of 2.64% (w/v) carboxylated beads were put into 1 mL of albumin solution in 50 mM MES buffer at pH 5.5 with concentration of 1, 0.75, 0.5, 0.25, and 0.1 µg mL^−1^, and incubated for 1 h at room temperature for the physical adsorption of albumin to the beads. Subsequently, the beads were washed with centrifugation for three times at 6000 r.p.m. for 3 min each, to get rid of the remaining unabsorbed albumin in the solution. The beads then were diluted to 3 µL for the experiment.

### Antibody against HSA conjugation on beads

Polyclonal rabbit anti-HSA (Abcam, ab34856) was diluted 80 × and conjugated on 1 µm PS-COOH beads surface via *N*-(3-dimethylaminopropyl)-*N*'-ethylcarbodiimide hydrochloride (EDC, Sigma Singapore E7750) and *N*-hydroxysuccinimide (NHS, Sigma Singapore 130672) coupling. Briefly, EDC and NHS was added to PS-COOH beads to activate the carboxyl groups and mixed under vortex for 1 h at 1650 r.p.m., at 4 °C. After activation, the beads were washed three times by centrifugation at 6000 r.p.m. for 5 mins. Before adding the antibody, the bead solution was subjected to probe sonication for 1 min, to ensure uniform dispersion of the beads. The antibody-beads solution was incubated for 3 h at 1650 r.p.m. vortexing at 4 °C.

### Antibody-conjugated beads-based detection of HSA on DLD

The conjugated beads were mixed with different concentration of HSA (Abcam) of 0.075, 0.05, 0.025, and 0.01 µg mL^−1^ for 1 h at room temperature and washed by centrifugation at 6000 r.p.m. for 3 min before putting in the sample inlet for DLD separation with 10 mM NaOH buffer. The device used was plasma-treated PDMS, which has been treated with pluronic F-127 for 30 min to prevent particle adhesion. The number of particles in the sub-channels were counted and plotted as the particle output distribution. The mean of the distribution would correspond to the apparent particle size in the DLD device. The LOD of the vesicle was then determined from the apparent diameter difference with the uncoated beads.

### Polymer vesicle preparation

Poly(butadiene-b-ethylene oxide) (PBd(1200)-PEO(600)) di-block co-polymer (P9089-BdEO, polymer source**)** was obtained to prepare vesicles, referred here as BD21, by film hydration method. Briefly, 5 mg of polymer was dissolved in 200 µl of chloroform, which was evaporated slowly by using stream of nitrogen in fume hood to make a thin film of polymer. To prepare dye labelled vesicles, Rhodamine B octadecyl ester perchlorate (RBOE, Sigma) dye was added in the polymer solution in chloroform before making the thin film. Polymer thin films (with and without RBOE) was vacuum dried for 4 h. To this, 1 mL of 1 × PBS (pH 7.2) was added and stirred overnight on magnetic stirrers (IKA-Werke multi-position, RT 15 Power, Germany) at 400 r.p.m. to make vesicles that was downsized by extrusion (6 times through 0.45 µm and 6 times through 0.22 µm filter) and dialysed to remove the free RBOE. Both BD21 and BD21-RBOE vesicles were characterized for size and surface charge by ZetaSizer (Malvern Instrument, UK) and transmission electron microscopy (JEOL 2010F transmission electron microscope from Jeol Ltd, Tokyo, Japan). To confirm the incorporation of RBOE dye in vesicles, fluorescence intensity spectra was measured by fluorescence spectroscopy at excitation of 533 nm.

### Reconstitution of membrane protein in polymer vesicles

The vesicle was solubilized using 50 µL of 10% Triton x-100. To this vesicle–detergent–micelle suspension, Aqp1 membrane protein (ab114210, Abcam) was added in 1:1 weight ratio to vesicle and incubated for 1 h at 4 °C. After this, 200 mg of Bio-Beads SM-2 was added for detergent removal accompanied with incorporation of Aqp1 in polymer vesicles. The Aqp1 reconstituted vesicle was characterized for vesicle size and fluorescence intensity by DLS and microplate reader, respectively. Furthermore, confirmation for the presence, functionality, and quantity of Aqp1 in vesicle after reconstitution was done by immunoassay by attaching them on PS micro-beads, osmotic permeability assay, and BCA assay, respectively.

### Polymer vesicles adsorption on beads

As prepared RBOE-BD21 vesicles were mixed with 1 µm-size PS beads and incubated at 1650 r.p.m. for 1 h. After this, the beads were washed three times by centrifugation at 6000 r.p.m. for 5 mins. The vesicles attachment on to the beads was characterized by the fluorescence intensity measurement at 533 nm excitation by Infinite M200 PRO multimode microplate reader (Tecan) and bright-field and fluorescence microscopy imaging.

### Antibody against Aqp1 conjugation on beads

Polyclonal rabbit anti-Aqp1 antibody against Aqp1 membrane protein (AQP001, Alomone lab) was diluted 20 × and conjugated on 1 µm PS-COOH beads surface via EDC/NHS coupling. Briefly, EDC/NHS was added to PS-COOH beads to activate the carboxyl groups and mixed under vortexing for 1 h at 1650 r.p.m., at 4 °C. After activation, the beads were washed three times by centrifugation at 6000 r.p.m. for 5 min. Before adding the antibody, the bead solution was subjected to probe sonication for 1 minute to ensure uniform dispersion of the beads. The antibody beads solution was incubated for 3 h at 1650 r.p.m. vortexing, at 4 °C. The conjugation of antibody to beads was confirmed by binding of mouse anti-rabbit antibody against rabbit anti-Aqp1 bound to Aqp1 protein.

### Beads based detection of Aqp1 vesicles in suspension

Before adding the Aqp1-vesicles for detection, antibody conjugated beads were incubated with blocker solution (1 × PBS solution of 1% bovine serum albumin with 0.01% of pluronic), at 700 r.p.m. for 2 h, room temperature to reduce the nonspecific binding. After blocking, antibody beads were washed thrice at 6000 r.p.m. for 5 min at 4 °C and mixed with vesicles with and without Aqp1 for incubation at 700 r.p.m. for 1 h, room temperature. Next, the beads were washed thrice at 6000 r.p.m. for 5 min and mixed with primary antibody (mouse anti-Aqp1, Abcam ab117970, 20 × dilution) at 700 r.p.m., 2 h, room temperature), washed thrice (6000 r.p.m. for 5 min at 4 °C) and mixed with secondary antibody (Alexa Fluor 647-conjugated goat anti-mouse IgG, Abcam ab150113, 20 × dilution). Finally, the beads were washed thrice at 6000 r.p.m. for 5 min at 4 °C before characterization using bright-field and fluorescence microscopy imaging.

### Beads-based detection of Aqp1 vesicles on DLD

The conjugated antibody is mixed with different concentration of vesicles with Aqp1 membrane protein and put in the sample inlet for separation with 0.1 × PBS as the buffer. The DLD device used was native PDMS device which has been treated with pluronic F-127 for 30 min to prevent particle adhesion. The number of particles in the sub-channels were counted and plotted as the particle output distribution. The mean of the distribution would correspond to the apparent particle size in the DLD device. The LOD of the vesicle is then determined from the apparent diameter difference with the uncoated beads.

### Experimental setup

The fluid flow in the microfluidic device was driven by output fluid extraction using a Chemyx syringe pump and a Hamilton 100 µl glass syringe. The input sample and buffer reservoirs were exposed to atmospheric pressure. This method facilitates rapid washing and change of buffer solutions. The experiment was visualised using an upright microscope and the particle flows for input and output regions of the device were captured using high speed Phantom M310 camera. The frame rates used were 50fps for the detection zone at × 100 magnification and 1000 fps for the high-speed imaging of individual bead motion within the DLD pillars at × 600 magnification. The number of particles flowing at different outlet sub-channels were counted and plotted as the particle output distribution for data analysis (Supplementary Note 3).

### Particle trajectory modelling

The computational modelling was performed using COMSOL Multiphysics 5.0. The geometry of the simulation was set as the actual system 1 device with 6 µm pillar, 4 µm gap, and 0.75° diameter with 250 µm s^−1^ velocity. The stokes flow module was used to get the velocity profile across the pillar and particle tracing of 1 µm diameter at different position relative to the pillar, which mimic the position tracking from the experiment, was used to get the trace of the particle over time with the time-dependent study.

### Data availability

The data that support the findings of this study are available from the corresponding author upon reasonable request.

## Electronic supplementary material

Supplementary Information(PDF 2409 kb)
