# Supplementary material for: Fluorescent label-free quantitative detection of nano-sized bioparticles using a pillar array
Source: Nat Commun. 2018 Mar 28;9:1254. doi: 10.1038/s41467-018-03596-z (PMC5871788; doi:10.1038/s41467-018-03596-z)
Supplement: Supplementary file 1 — Supplementary Information(PDF 2409 kb) [file 41467_2018_3596_MOESM1_ESM.pdf]

Supplementary Information

Fluorescent Label-free Quantitative Detection of Nano-sized Bioparticles Using A Pillar Array

Kerwin Kwek Zeming[1], Thoriq Salafi[1,2], Swati Shikha[1], Yong Zhang[1,2,*]

[1] *Department of Biomedical Engineering, National University of Singapore, Singapore 117575*

[2] *NUS Graduate School for Integrative Sciences and Engineering, National University of Singapore, Singapore 117456*

[*]Corresponding author:

Prof. Yong Zhang

Department of Biomedical Engineering

Faculty of Engineering, Block E4 #04-08

National University of Singapore

4 Engineering Drive 3, Singapore 117583

Phone: +65-65164871

Fax: +65-68723069

Email: biezy@nus.edu.sg

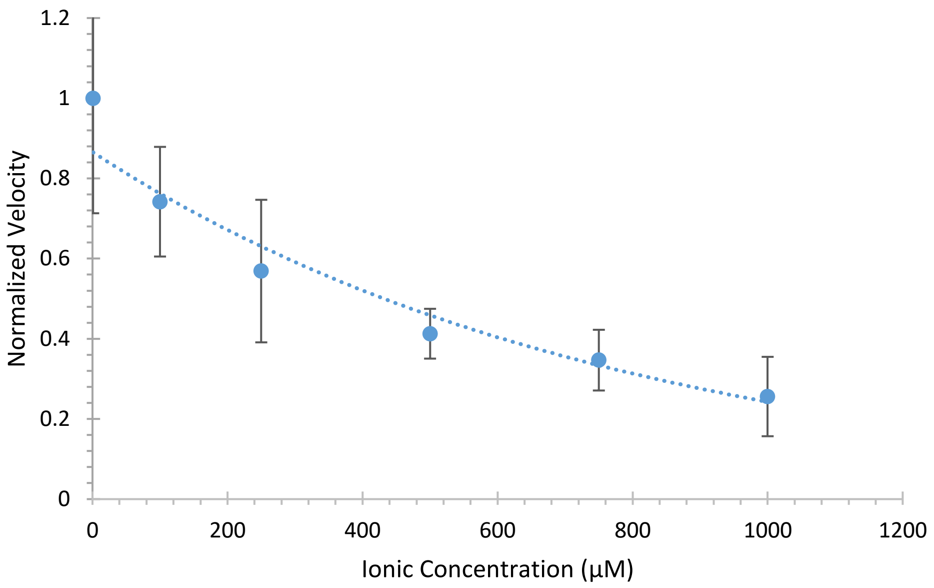

Supplementary Figure 1. The normalised flow velocity of 1 µm PS beads at various ionic concentration and at the same region in the DLD device. The average flow velocity is tracked using highspeed camera capturing at frame rates of 1000 fps. The time taken for beads to flow over a fixed length was measured. The speeds were normalised to flow velocity of beads in DI water. Error bars are s.d. from 5 different beads' velocity

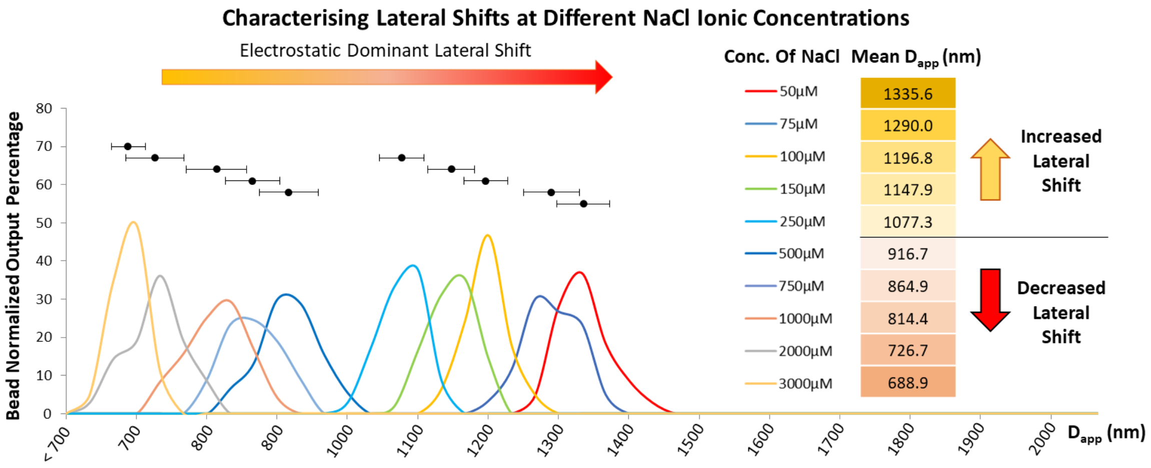

Supplementary Figure 2. Various output spectrum of 1µm PS beads separation in DLD device at various ionic concentration of NaCl. The output separation spectrum and D_{app} of 1µm PS beads at different ionic concentrations of NaCl. D_{app} describes the apparent size of the 1µm bead. An increasing electrostatic repulsion correlates to an increase in D_{app} while a decrease in electrostatic interactions reduces the lateral shift. All data point comprises of the distribution of at least 50 beads with the error bar representing the s.d. of the calculated D_{app}.

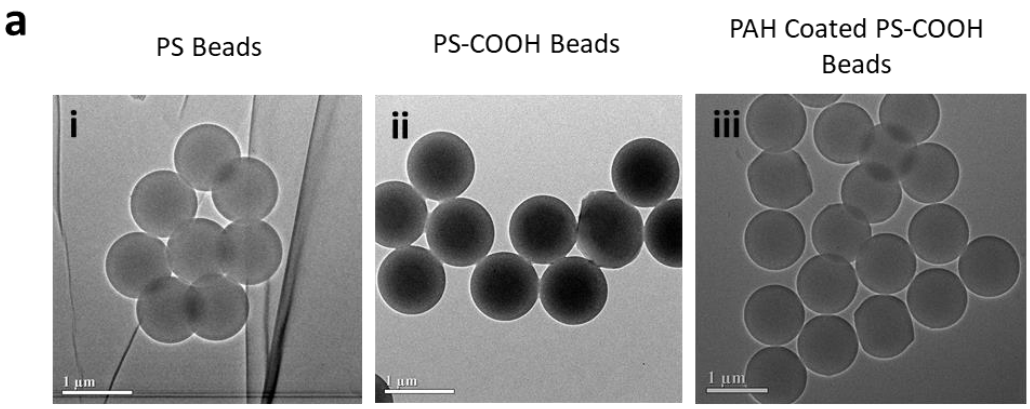

b

Measurement	PS Beads	PS-COOH Beads	PAH-coated PS-COOH Beads
TEM (nm)	977 ± 4.0	991 ± 4.3	985 ± 4.1
DLS (nm)	1071 ± 34	1135 ± 23	1095 ± 46
Zeta @ DI H_2O (mV)	-21.4 ± 0.24	-29.5 ± 0.30	43.2 ± 1.29

Supplementary Figure 3. Size and charge characterisation of 1µm beads. (a) TEM images of the different bead surfaces. Scale bar is 1 µm **(b)** Using TEM and DLS, the physical and hydrodynamic size of the beads and their relative size difference were measured. The Zeta potential measurement detects the difference in surface charge and stability of the colloidal system. Error bars are s.d. with n=5

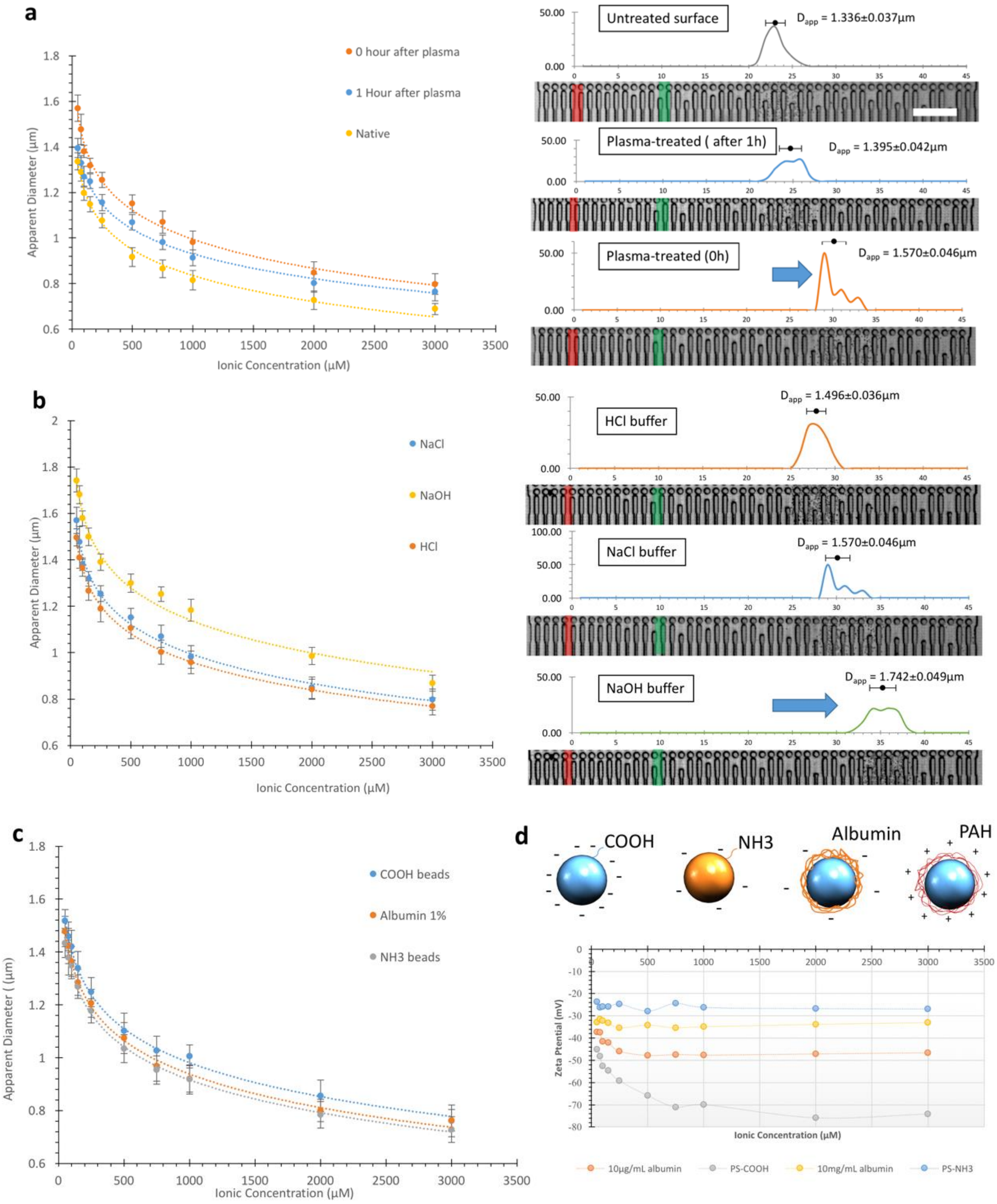

Supplementary Figure 4. Separation of particles in different surface treatment, pH buffer, and different type of beads in DLD system 1. (a) Separation of PS beads after plasma treatment, 1 hour after air exposure and native PDMS in DLD system 1. The plasma treatment oxidizes the -CH_3 group to -OH on PDMS surface, therefore the surface charge is more negative which results in larger repulsion force and higher Dapp (scale bar = 50 µm) **(b)** The response of different pH buffer in the separation of PS beads in plasma treated device. It can be seen that the alkaline pH buffer improves the separation due to the Ionization of OH group at high pH. **(c)** The separation of PS-COOH, PS-NH_2 and albumin coated beads. The albumin coated beads and NH_2 beads seems to have less separation compared to COOH beads although there is large overlap among the separation spectrum. Error bars are s.d. from the distribution of at least 50 beads.**(d)** Zeta potential of different beads used in the experiments in NaCl.

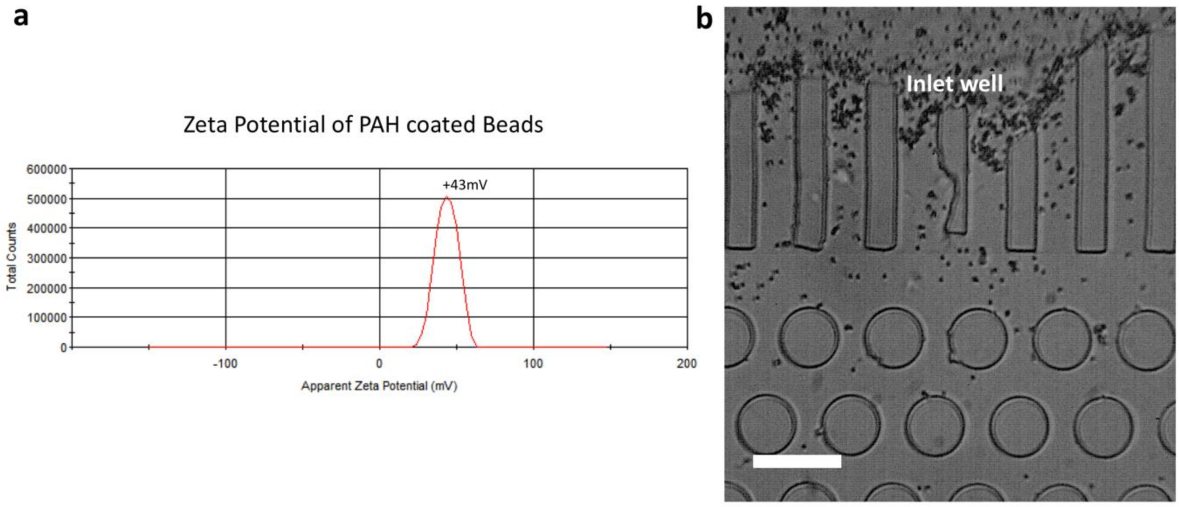

Supplementary Figure 5. Clogging of PAH-coated beads in the inlet reservoir. The PAH coated beads were clogged in the inlet reservoir due to the electrostatics attraction force between the positively charge PAH coated beads (+43mV in DI water) with the negatively charged PDMS surface (*scale bar = 20 µm)*

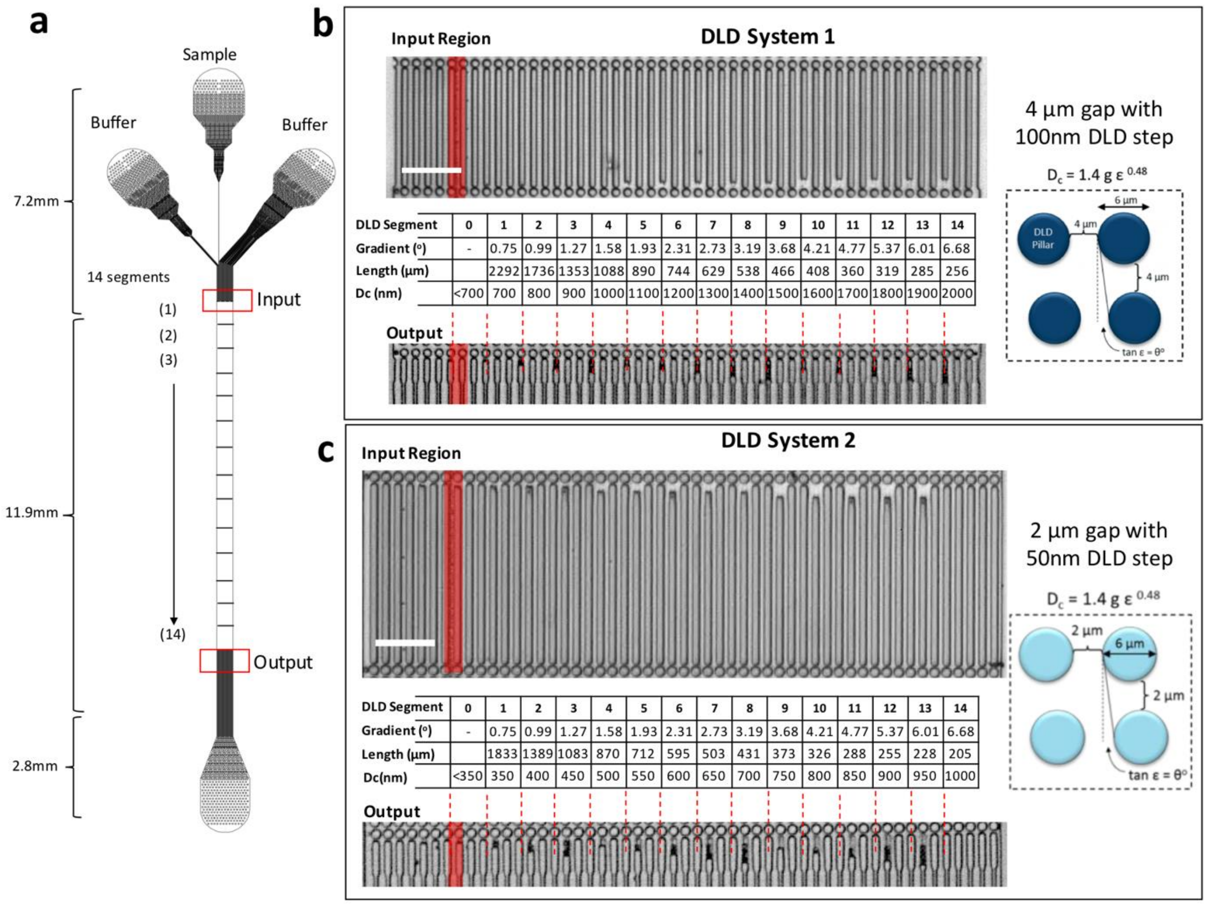

Supplementary Figure 6. Device design. The device used are chirped DLD array with the 100nm resolution with gap size of 4µm for system 1 (DLD-S1) and 50nm resolution with 2µm gap for system 2 (DLD-S2) device. **(a)** The device has three inlets, with the middle inlet as the sample inlet while side inlets for buffers, and one outlet for the tubing for generation of negative flow rate. **(b)** The device has 14 segments with different angle and length with Dc ranging from 700 to 2000nm for system 1 with 100nm resolution for each set of angles (scale bar = 50 µm). **(c)** For system 2, the resolution is 50nm with the Dc ranging from 350nm to 1000nm. The inlet positions are shown in red and the corresponding separation Dc is shown in the table (scale bar = 40 µm).

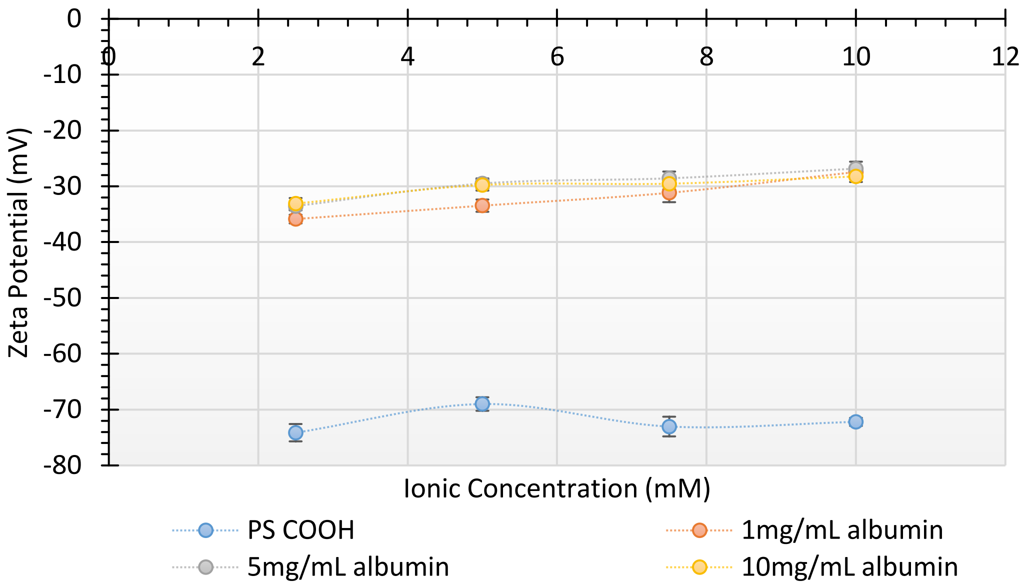

Supplementary Figure 7. The zeta potential of uncoated and albumin coated beads in NaCl solution. Shows the zeta potential of uncoated PS-COOH beads and albumin coated beads of 1, 5, and 10 mg mL^{-1} albumin. It can be seen that the zeta potential of the coated beads is less negative compared to the uncoated beads due to the shielding of the beads surface charge. The error bars are s.d. with n=3.

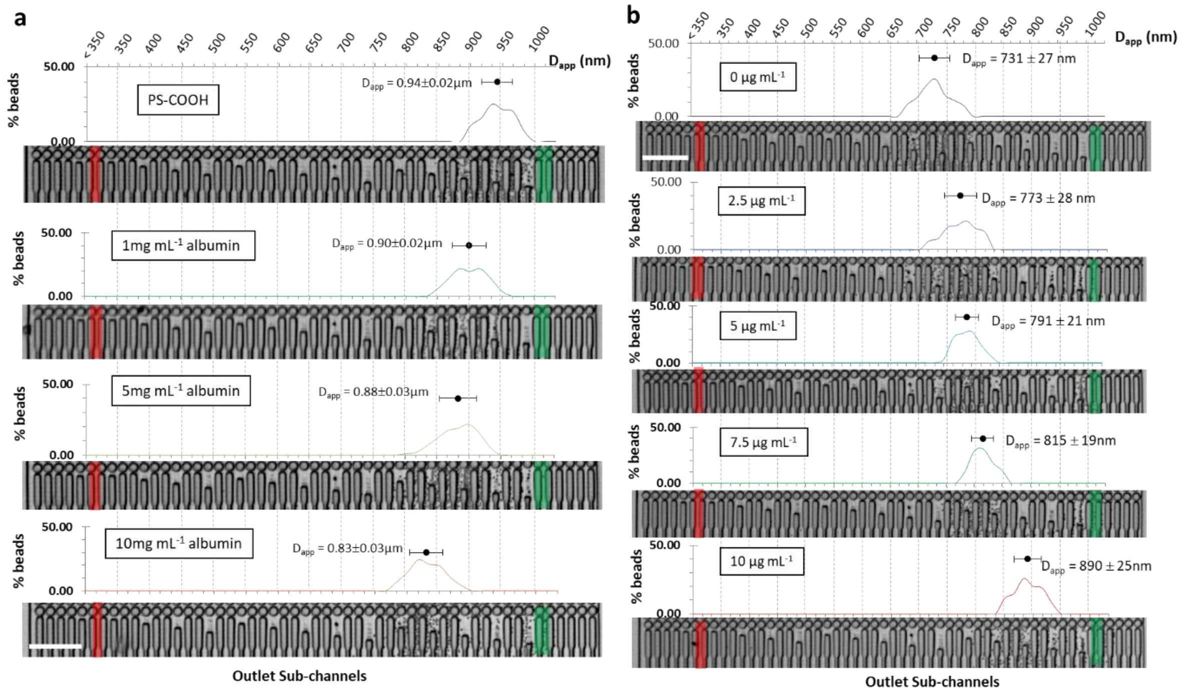

Supplementary Figure 8. DLD output spectrums of D_{app} shifts measurements for albumin detection. The figure shows D_{app} of different protein concentration of coated beads. (a) the detection of albumin using NaCl as the electrostatic charge modulation condition shows that increasing albumin adsorption reduces the Dapp (scale bar = 40 µm). (b) shows that the converse is true for NaOH pH 12 solution where increasing the adsorption of proteins increases the D_{app} size due to the increase in negative charge groups induced by NaOH exposure. The amount of proteins to be detected is also reduced to the µg ml^{-1} range (scale bar = 40 µm).

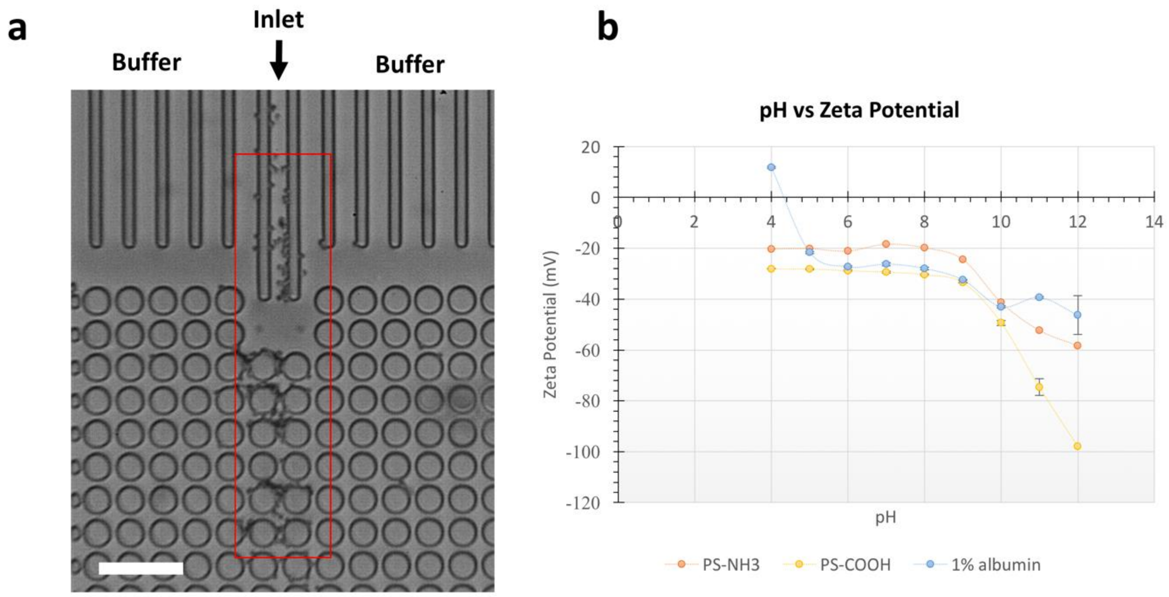

Supplementary Figure 9. Clogging of albumin coated beads in 1mM HCl buffer. (a) Albumin coated beads were clogged in 1 mM HCl buffer due to the switching of the albumin charge from negative to positive at pH 4.7, which results in the particle deposition due to electrostatics attraction of the particle with PDMS device (scale bar = 20 µm). (b) Zeta potential of different beads in different pH buffer, error bars are s.d. with n=3.

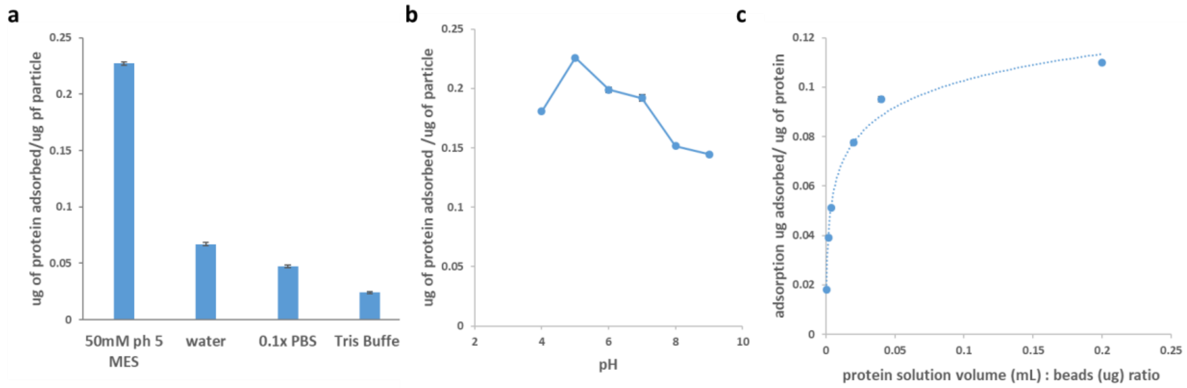

Supplementary Figure 10. Protein adsorption optimisation (a) Optimisation of adsorption of proteins in various media. (b) Adsorption of proteins on bead substrate suspended in 50mM 2-(N-morpholino) ethanesulfonic acid (MES) buffer at various pH. (c) Adsorption of proteins of fixed mass in various MES solution volume with respect to the amount of beads. the error bars are s.d. from n=3.

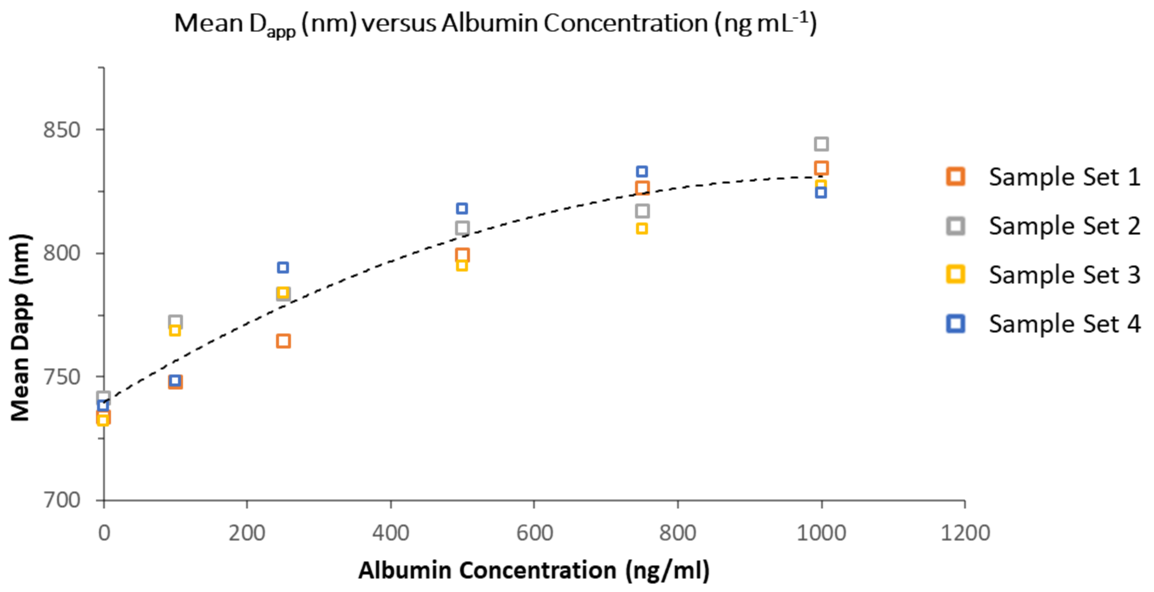

Supplementary Figure 11. Individual plots of mean D_{app} versus albumin concentration. Albumin of concentration 0, 100, 250, 500, 750, 1000 ng ml^{-1} were tested and the mean D_{app} for each sample set was measured. The black dash lines show the mean plots curve.

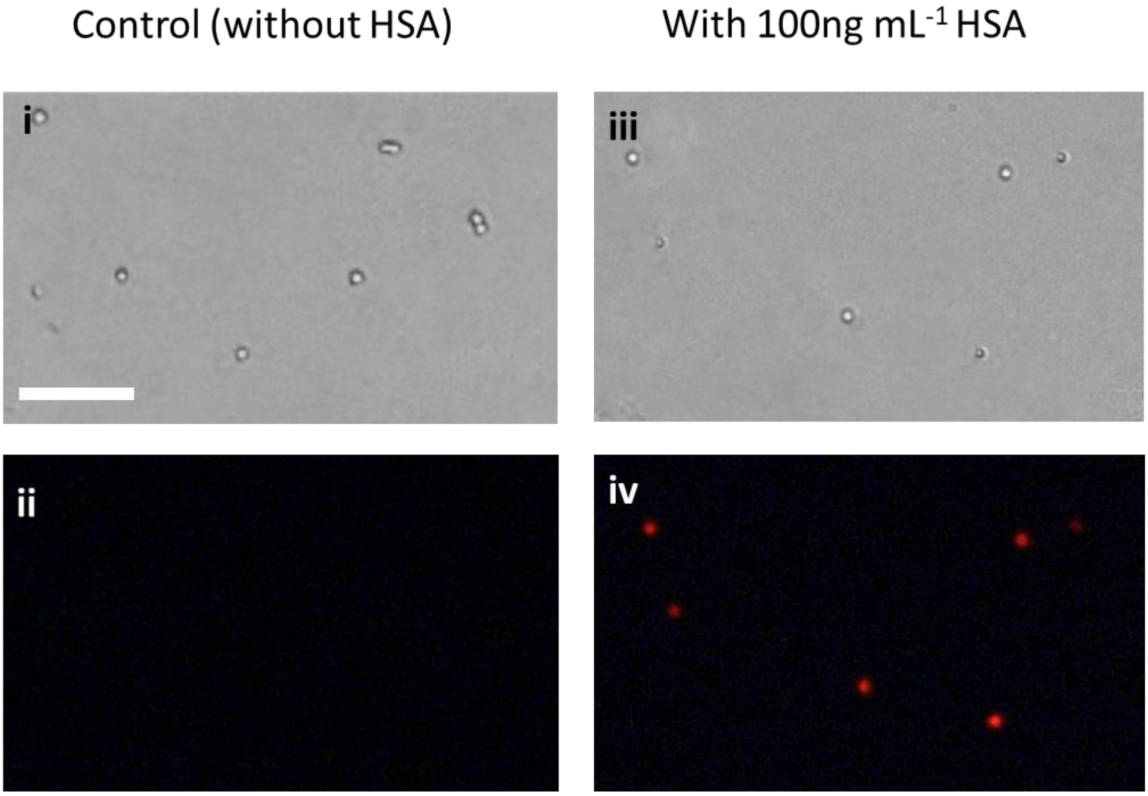

Supplementary Figure 12. Confirmation of HSA binding to antibody conjugated bead substrate. To ensure antibody conjugated beads remain functional to HSA binding, we performed a binding step using 100 ng ml^{-1} HSA sample and performed a secondary antibody binding step coupled with fluorescence to the bound HSA on bead surface (a-b) control and (c-d) 100ng ml^{-1} HSA (scale bar = 10 µm).

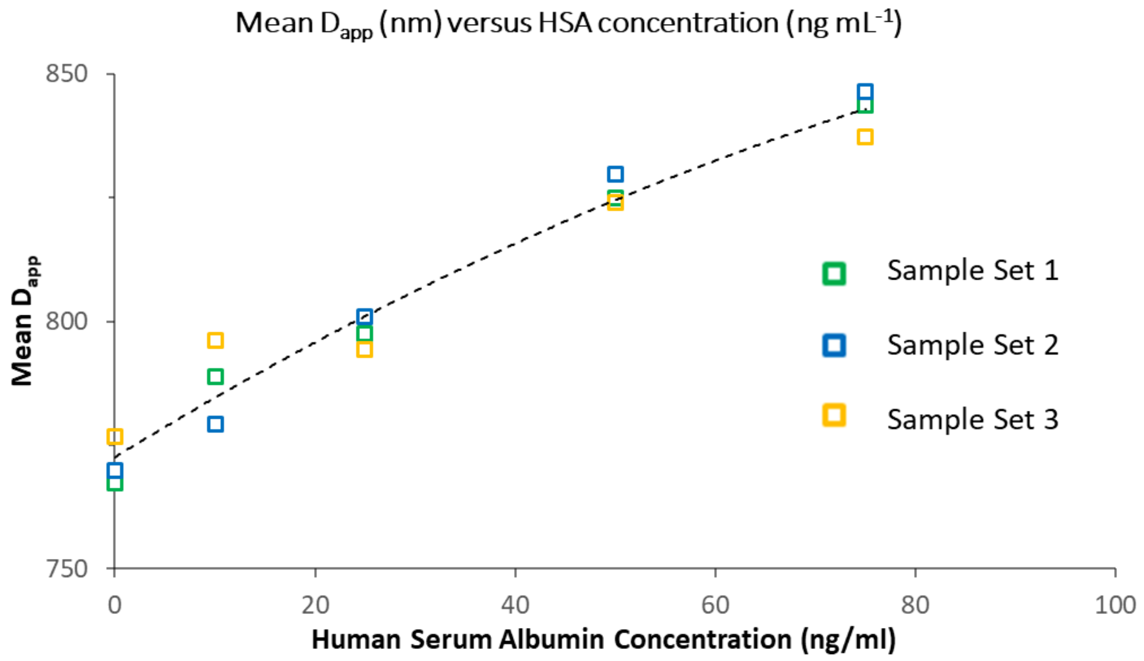

Supplementary Figure 13. Individual plots of mean D_{app} versus HSA concentration. Albumin of concentration 0, 10, 250, 50 and 75 ng ml^{-1} were tested using beads conjugated with HSA antibodies. The mean D_{app} for each sample set was measured. The black dash lines show the mean plots curve.

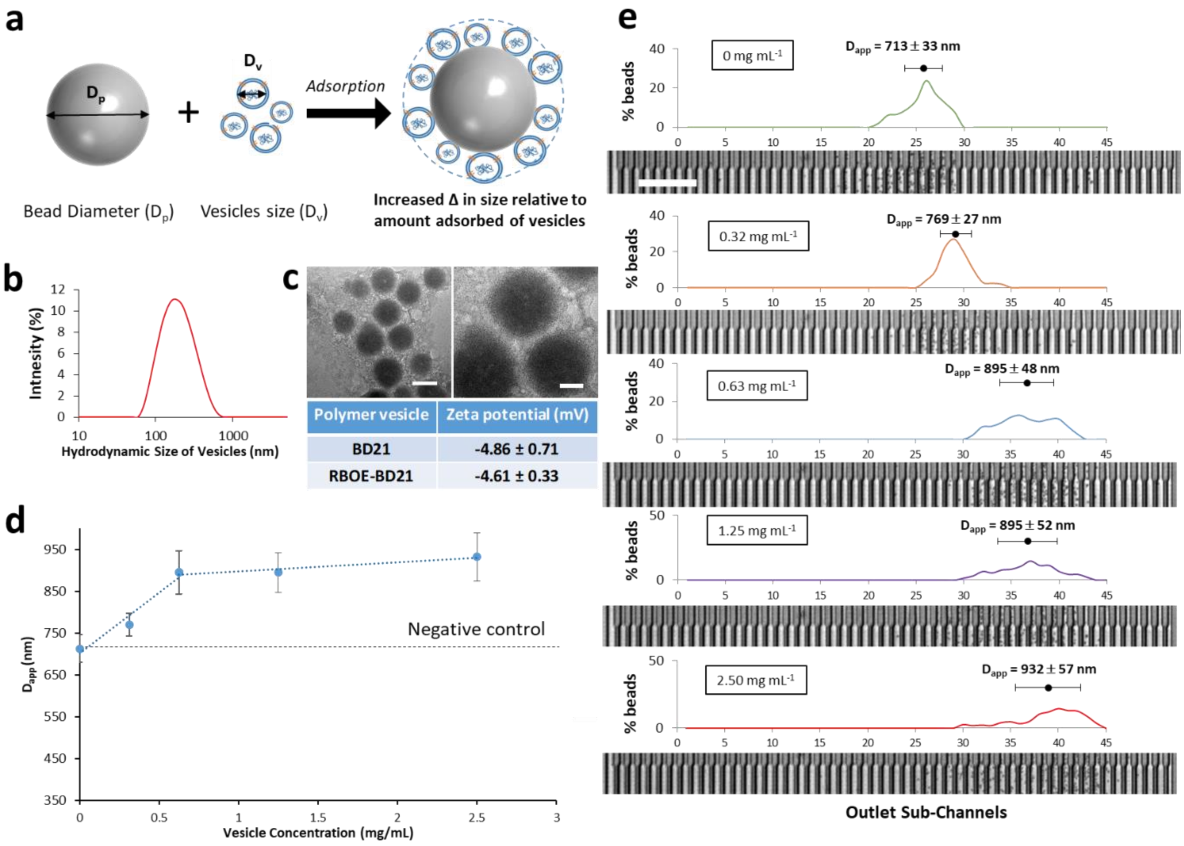

Supplementary Figure 14. Detection of nano-vesicles via change in physical size relative to the amount of adsorbed vesicles. (a) The schematic showing the change in physical size of beads due to adsorption of nano-vesicles. (b) and (c) show the DLS, TEM and zeta-potential measurements of vesicle size in the colloidal suspension respectively (left scale bar = 100 nm, right scale bar = 50 nm). (d) shows the corresponding D_{app} of PS-COOH 1 μm beads coated with different concentration of vesicles and (e) the corresponding DLD spectrum. The buffer media used here is 0.1x PBS solution (scale bar = 40 μm). All data point comprises of the distribution of at least 50 beads with the error bar representing the s.d. of the calculated D_{app}.

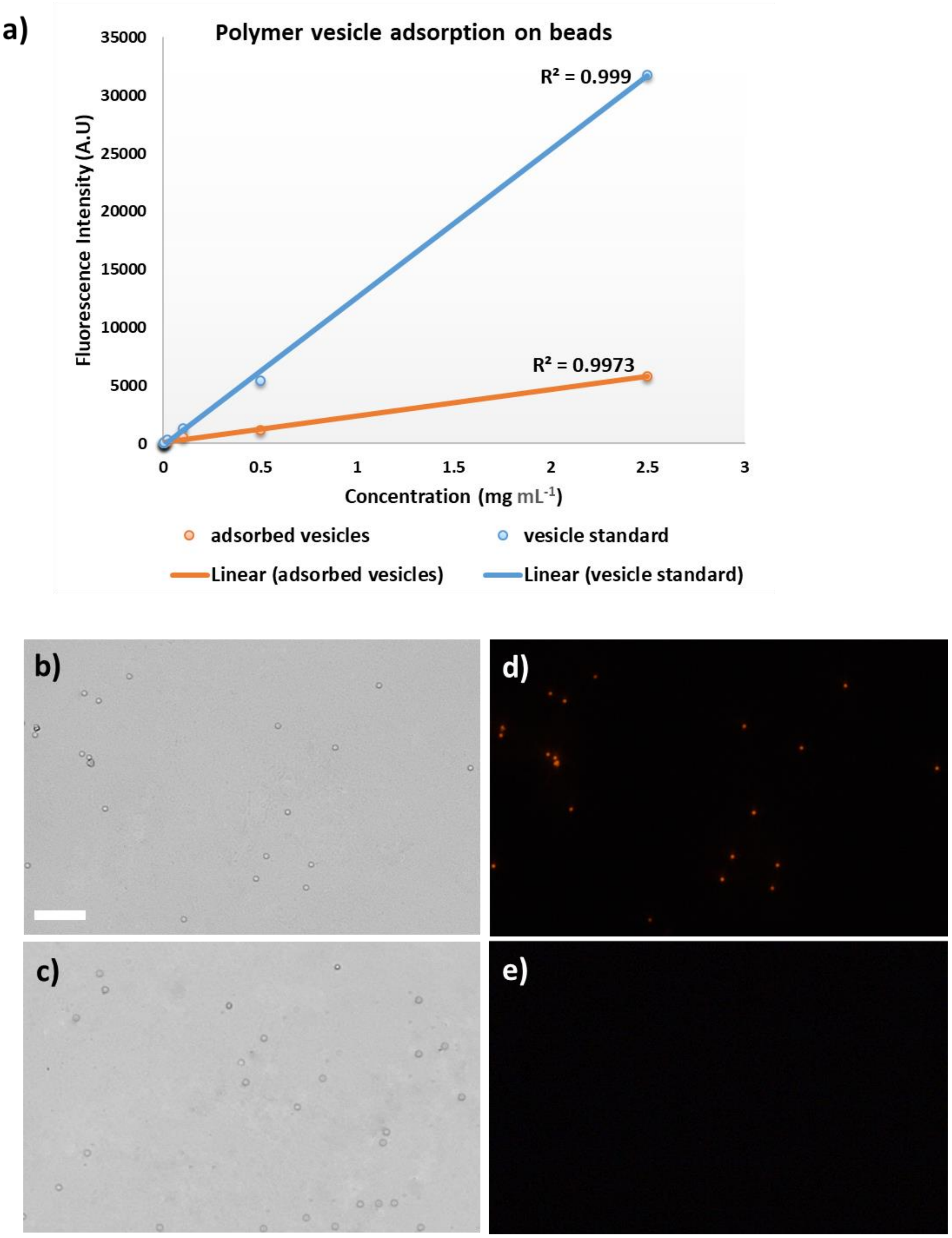

Supplementary Figure 15. Physical adsorption of polymer vesicles on beads. RBOE-BD21 vesicle adsoprtion on PS beads **(a)** Concentration dependent fluorescence intensity of polymer veiscle adsorbed on beads. Bright-field imaging of **(b)** vesicles on beads (scale bar = 10 µm) **(c)** control (no vesicles), at 40x magnification, 10ms exposure and 4x gain. Fluorescence imaging of **(d)** vesicles on beads and **(e)** control (no vesicles) on beads, at 40x magnification, 800 ms exposure and 4x gain.

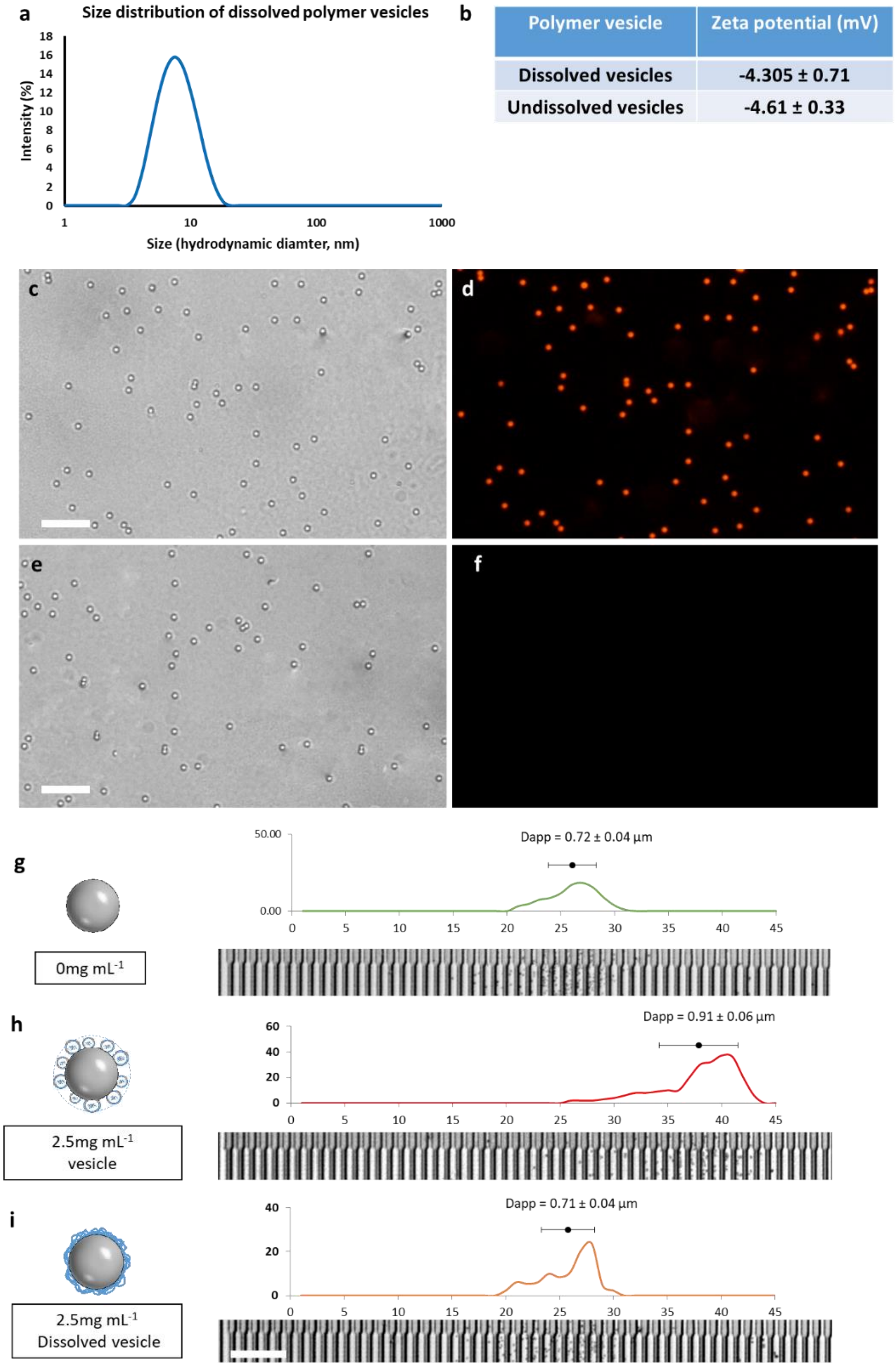

Supplementary Figure 16. Analysis of separation and detection mechanism: size based or charge based. Adsorption of dissolved vesicles (size of ~8 nm) on beads. **(a)** DLS data showing size distribution of dissolved vesicles **(b)** Zeta potential of dissolved vesicles. Bright-field images of **(c)** dissolved vesicles adsorbed on beads (scale bar = 10 µm) **(e)** control beads (no vesicles), at 40x magnification, 10ms exposure and 4x gain. Fluorescence imaging of **(d)** dissolved vesicles on beads and **(f)** control beads (no vesicles), at 40x magnification, 800 ms exposure and 4x gain **(g)** separation of control beads **(h)** vesicle increase the size of the beads to up to +200nm and the separation in DLD reflects this change, **(i)** while the dissolved vesicle with size ~8nm does not have change in the displacement (scale bar = 40 µm). All data point comprises of the distribution of at least 50 beads with the error bar representing the s.d. of the calculated D_{app}.

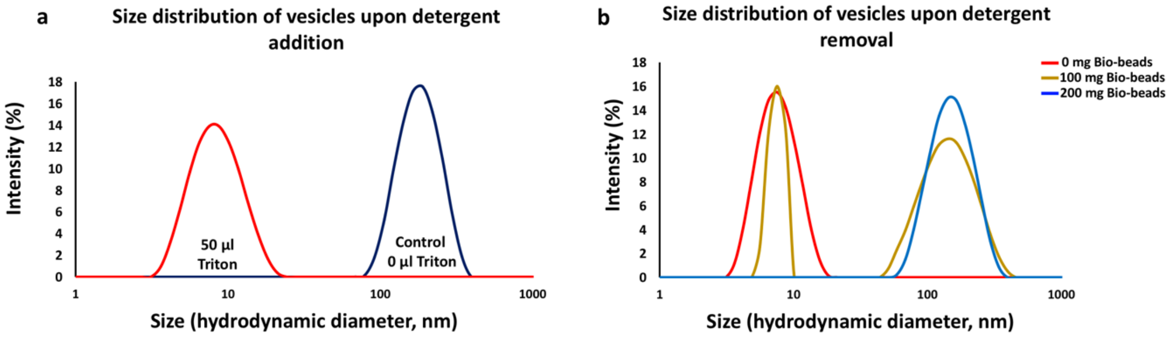

Supplementary Figure 17. Reconstitution conditions for incorporating membrane protein in BD21 vesicle. Detergent for vesicle dissolution. (a) DLS showing size distribution of BD21 for 0 µl (control, 219 ± 123nm) and 50 µl of 10% triton x-100. Vesicle sample with 50 µL of detergent was dissolved to a size of 8.8 ± 3.2 nm (amounting to 95% of total intensity), thereby suggesting that most of the vesicles have been solubilized into polymer-detergent micelles. Bio-beads for removal of detergent from detergent-polymer micelle suspension. **(b)** DLS showing size distribution of samples after adding 0 mg (control), 100 mg and 200 mg of bio-beads. Compared to dissolved vesicles with no Bio-Beads added, sample treated with 200 mg of Bio-Beads showed the size of reconstituted vesicle to be 150.9 ± 70.91 nm that accounted for 95.7% of total intensity. The smaller size of the reconstituted vesicles compared to the original size of vesicles could be due to a faster rate of detergent removal.

a

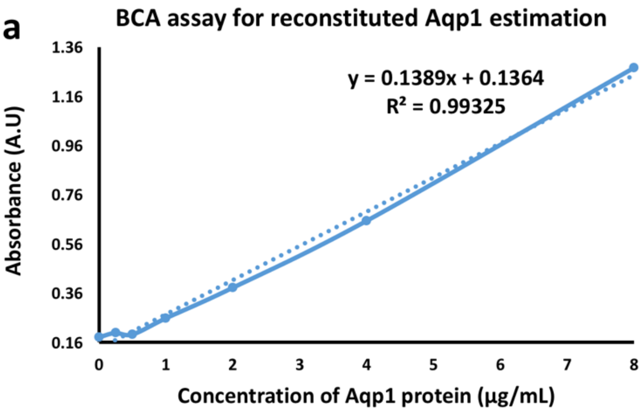

b

Sample	Concentration (µg/mL)
BD21-Aqp1	0.37 µg/mL

Supplementary Figure 18. BCA assay for quantification of Aqp1 membrane protein in BD21. (a) Standard curve (0 to 8 µg mL^{-1}) **(b)** Amount of Aqp1 membrane protein in BD21 polymer vesicles after reconstitution. Known concentrations of Aqp1 were used for preparing the standard curve, based on which the Aqp1 amount in reconstituted vesicle was found to be to be 0.37 µg mL^{-1}.

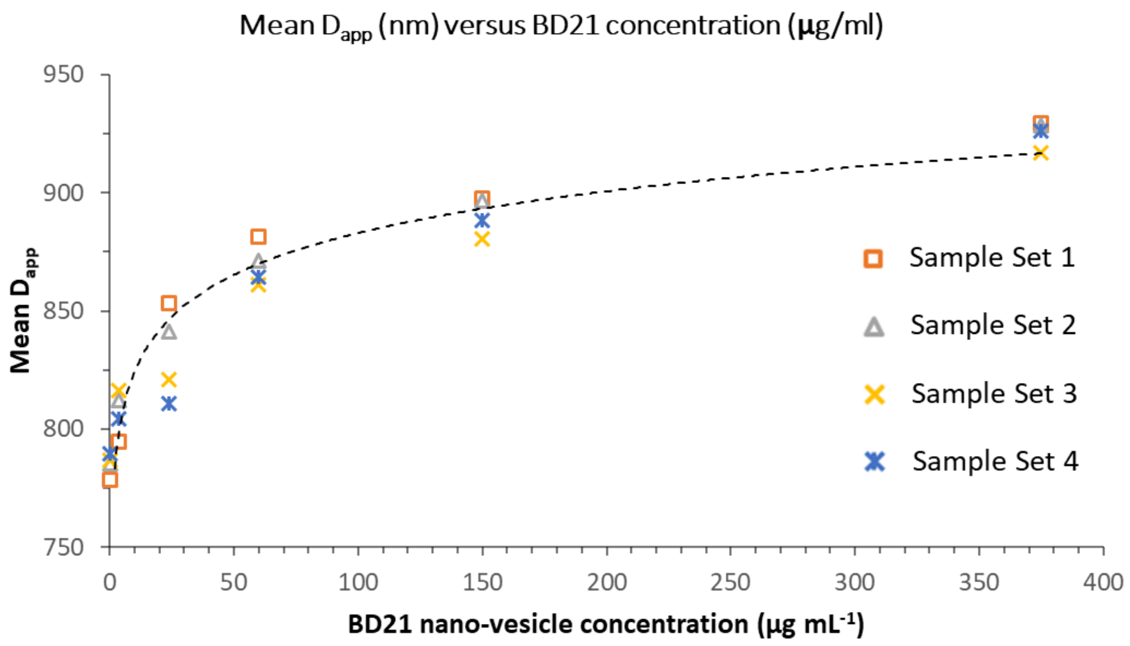

Supplementary Figure 19. Individual plots of mean D_{app} versus BD21 nano-vesicles concentration. BD21 of concentration 0, 3.75, 24, 60, 150, 375 µg ml^{-1} were tested and the mean D_{app} for each sample set was measured. The black dash lines show the mean plots curve.

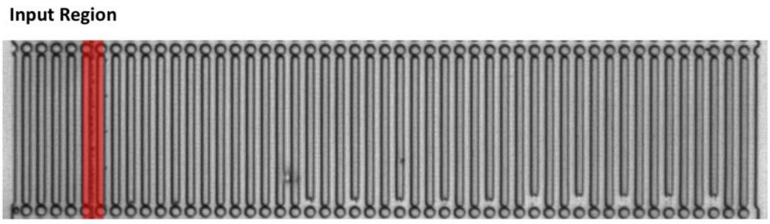

DLD Segment	0	1	2	3	4	5	6	7	8	9	10	11	12	13	14
Gradient (°)	-	0.75	0.99	1.27	1.58	1.93	2.31	2.73	3.19	3.68	4.21	4.77	5.37	6.01	6.68
Length (μm)		2292	1736	1353	1088	890	744	629	538	466	408	360	319	285	256
Dc (nm)	<700	700	800	900	1000	1100	1200	1300	1400	1500	1600	1700	1800	1900	2000

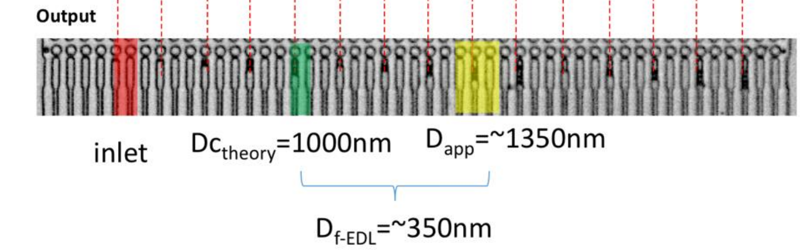

Supplementary Figure 20. Concept of apparent Diameter in nano-regime DLD. DLD uses pillar gap and angle to create the empirical critical diameter formula for particle separation of $Dc_{formula} = 1.4G\tan\theta^{0.48}$. However, in nano-regime DLD, the particle separation is deviated from this formula which we called the apparent diameter D_{app}. The deviation of this apparent diameter from the empirical DLD formula is due to the additional distance from the electric double layer force (D_{f-EDL}). Hence, the apparent diameter is $D_{app} = Dc_{formula} + D_{f-EDL}$

Sample		Average hydrodynamic size (nm)	Amount of shrinking
BD21 only	Before osmotic shock	140.6 ± 37.05	5.69%
	After osmotic shock	132.6 ± 24.61	
BD21-Aqp1	Before osmotic shock	145.4 ± 42.79	41.91%
	After osmotic shock	84.5 ± 17.51	

Supplementary Table 1. Functionality of Aqp1 after reconstitution. DLS data showing hydrodynamic size of BD21 vesicle to check the water permeability with and without Aqp1 membrane protein, in response to sucrose osmotic shock. The DLS data revealed that the size of vesicles without Aqp1 decreased only by 5.69%, whereas vesicle with Aqp1 was shrunk up to 41.91% after osmotic shock, thereby suggesting the role of Aqp1. It could be inferred that the water permeability functionality of membrane protein was preserved after reconstitution.

Supplementary Note 1

DLD resolution and factors affecting the limits of detection

The device DLD-S1 has a step resolution of 100 nm which is the same step resolution of the original work by Huang et al[1]. Thus, the minimum resolution of detection is around 20nm.

However, we have designed a DLD device with a 50nm step resolution and based on analysis using multiple devices and independent experiments, we found the average standard deviation of mean to be around 10nm. Thus, it is safe to assume that the resolution is more than 10nm and less than 20nm.

Moreover, our DLD devices have 14 segments with 3 periods of DLD arrays per segment (Supplementary Fig. 1). This would mean that for each step resolution, we could divide it by 3 and resulting in a resolvable quasi-resolution of 34 nm for DLD-S1 and 17 nm for DLD-S2. The input sample stream is sandwiched by two buffer streams to a width of a single input channel.

These are the factors affecting the limits of detection.
(1) Carrier bead size variance: the carrier bead we are using has a CV of <3% which will definitely impact the resolution of detection by 10nm. Thus, to overcome these, the electrostatic difference has to be enhanced to overcome these limits.

(2) Diffusion: diffusion will definitely play a role. But at these flow velocities and 1 micron size particle, the diffusive effects are less dominant compared to the DLD effects (Cite the nature nanotech paper study on diffusion in DLD).

(3) Particle concentration: lastly, particle-particle interaction may cause decrease of efficiency and thus reduce the resolution.

Supplementary Note 2

Label-free detection of vesicle with DLD

The detection of EVs and its membrane proteins is crucial for disease diagnosis. Here, bead-based detection of vesicle on a DLD device based on size differences of beads with and without vesicles was proposed.

Owing to advantages of mechanical stability and membrane tunability, polymer vesicles were chosen over lipid vesicles for incorporating the membrane proteins for the current study.[2-4] Vesicles with a size range 131.64 ± 31.3 nm were prepared and characterised for size, shape and surface charge (Supplementary Table 1). These vesicles were physically adsorbed onto the surfaces of 1.0 µm polystyrene beads and detected using a DLD device based on increase in size owing to vesicles attachment as can be seen in Supplementary Figure 15. The vesicle coated beads of 2.5 mg mL^{-1} concentration were found to have enhanced displacement for average of more than 200 nm as compared to the uncoated beads (Supplementary Figure 15d and 15e). Furthermore, the distribution of the beads was observed to be larger in sub channels due to the uneven coating and vesicle size range. After this, different concentrations of vesicles on beads were detected in DLD device and the detection limit of vesicle coated beads was found to be 0.32 mg mL^{-1}, at which the displacement increases for size increase more than 50 nm. It is confirmed that the displacement is driven by change in size instead of charge by using dissolved vesicles as a control (Supplementary Figure 17 and 18).

Supplementary Note 3

Data analysis from the lateral displacement of the beads

For all the DLD experiments, the beads displacement on the output region was captured using high speed camera. The beads were then counted and "binned" into their respective output channel number. The normalized frequency distribution curve with bead count (n) ranging from 50 to 250 were plotted and the apparent diameter (D_{app}) was obtained from the mean of the bead distribution, with the standard deviation of:

$$\text{std} = \sqrt{\frac{\sum f(x-\overline{x})^2}{n}}$$

Where f represents the number of beads per "bin" and x represent the position of the bin and $\overline{x}$ the mean of the distribution (D_{app}) and n is the total number of beads counted. For all the calibration and optimization works, this method was used to analyse the performance of the device.

For more sensitive detection i.e. albumin detection (100 ng/ml – 1000ng /ml), Human Serum Albumin detection (10ng/ml – 75ng/ml) and Vesicle detection (3.75 – 375 µg/ml), the experiments were repeated for 3 to 4 times, and each D_{app} of the replications was then averaged to obtain the $\overline{\overline{D_{app}}}$, mean of mean D_{app}, for the datasets. The two sample independent t-tests were then performed for each concentration group for which the error bar is the sample standard deviation of the data plots of grouped mean D_{app}.

Supplementary References

1 Huang, L. R., Cox, E. C., Austin, R. H. & Sturm, J. C. Continuous particle separation through deterministic lateral displacement. *Science* **304**, 987-990, (2004).

2 Discher, B. M. *et al.* Polymersomes: tough vesicles made from diblock copolymers. *Science* **284**, 1143-1146 (1999).

3 Christian, D. A. *et al.* Spotted vesicles, striped micelles and Janus assemblies induced by ligand binding. *Nature Materials* **8**, 843-849 (2009).

4 Tanner, P. *et al.* Polymeric vesicles: from drug carriers to nanoreactors and artificial organelles. *Accounts of Chemical Research* **44**, 1039-1049 (2011).
